# Supplementary material for: Selective and stable CO2 electroreduction at high rates via control of local H2O/CO2 ratio
Source: Nat Commun. 2024 Jul 13;15:5893. doi: 10.1038/s41467-024-50269-1 (PMC11246503; doi:10.1038/s41467-024-50269-1)
Supplement: Supplementary file 1 — Supplementary Information [file 41467_2024_50269_MOESM1_ESM.pdf]

Selective and Stable CO_2 Electroreduction at High rates *via* Control of Local H_2O/CO_2 Ratio

Junmei Chen[1, †], Haoran Qiu[1, 2, †], Yilin Zhao[1, †], Haozhou Yang[1], Lei Fan[1], Zhihe Liu[1], ShiBo Xi[3], Guangtai Zheng[1], Jiayi Chen[1], Lei Chen[1], Ya Liu[2], Liejin Guo[2], Lei Wang[1, 4*]

[1] Department of Chemical and Biomolecular Engineering, National University of Singapore, Engineering Drive 4, Singapore 117585, Singapore

[2] International Research Center for Renewable Energy, State Key Laboratory of Multiphase Flow, Xi'an Jiaotong University, Xi'an, Shaanxi 710049, China

[3] Institute of Sustainability for Chemicals, Energy & Environment, A*STAR, 1 Pesek Rd, 627833, Singapore

[4] Centre for Hydrogen Innovations, National University of Singapore, 1 Engineering Drive 3, 117585, Singapore

[†] These authors contributed to the work equally

E-mail: wanglei8@nus.edu.sg

Table of Contents

Supplementary Figures **7**

Supplementary Fig. 1. Performance comparison of PTFE/Cu and Nafion/Cu at the same current densities.. 7

Supplementary Fig. 2. SEM images of polymer/Cu 8

Supplementary Fig. 3. Contact angles polymer/Cu. 9

Supplementary Fig. 4. Water uptake ability comparison of polymer membranes. 10

Supplementary Fig. 5. Flow-cell schematic. 11

COMSOL simulation **12**

Physical model 12

Supplementary Fig. 6. Schematic of modeling framework and boundary conditions. 13

Governing equations 14

Transport of species in the EL and PL 14

Acid-Base Equilibria. 17

Charge transfer reactions on the cathode. 18

Size reduction with x-coordinate transformation. 18

Supplementary Fig. 7. Schematic of the dimensions of each domain in the simulation model. 19

Supplementary Fig. 8. Modeled local species profile with varied polymer thickness and H_2O/CO_2 ratio.. 20

Supplementary Fig. 9. CO_2 concentration distribution and streamline distribution within the polymer layer at –1.426 V vs. SHE 21

Supplementary Fig. 10. Modeled local species profile with varied polymer porosity and H_2O/CO_2 ratio.. 22

Supplementary Fig. 11. Influence of the pore size of the catalyst layer. 23

Supplementary Fig. 12. SEM images of polymer/Cu GDEs. 25

Supplementary Fig. 13. CO_2 adsorption isotherms of polymer membranes under varied pressure. 26

Supplementary Fig. 14. Photograph of polymer membranes.. 27

Supplementary Fig. 15. Cu K-edge spectra of the five polymer/Cu catalysts.... 28

Supplementary Fig. 16. Cu LMM Auger spectra of Cu and polymer/Cu. 29

Supplementary Fig. 17. XRD pattern of polymer/Cu and initial Cu. 30

Supplementary Fig. 18. TEM and cross-section characterizations for PT/Cu, PCR/Cu and PT95/Cu GDEs. 31

Supplementary Fig. 19. SEM images of Cu with different polymer coatings.... 32

Supplementary Fig. 20. TEM images of Cu NP with different polymer coatings.. 33

Supplementary Fig. 21. Tested and simulated polarization curves comparison of polymer/Cu GDEs. 34

Supplementary Fig. 22. Faradic efficiencies for CO_2R products. 35

Supplementary Fig. 23. H_2 activity comparison of FE versus current densities. 36

Supplementary Fig. 24. C_{2+} cathodic energy efficiency comparison for polymer/Cu GDEs. 37

Supplementary Fig. 25. Correlation between regulated local H_2O/CO_2 ratio and the performance of C_{2+} products from CO_2R with five polymer/Cu GDEs. 38

Supplementary Fig. 26. Cathodic potential comparison for five polymer/Cu GDEs. 39

Supplementary Fig. 27. CO FE comparison for five polymer/Cu GDEs.. 40

Supplementary Fig. 28. CO partial current density comparison at the same cathodic potentials. 41

Supplementary Fig. 29. ECSA comparison. 42

Supplementary Fig. 30. Post contact angles.. 43

Supplementary Fig. 31. Visualization of the electrowetting effect. 44

Supplementary Fig. 32. Post gas bubble adhesion behaviors. 45

Supplementary Fig. 33. Post gas bubble adhesion behaviors at extended current density and time. 46

Supplementary Fig. 34. Post cross-sectional scanning electron microscopy and energy-dispersive X-ray spectroscopy elemental mapping images. 47

Supplementary Fig. 35. Stability of PT/Cu and Nafion/Cu at −1 A cm^{-2} in 1 M KOH.........48
Supplementary Fig. 36. Post SEM for different polymer/Cu.........49
Supplementary Fig. 37. CO_2 partial pressure experiment for PT/Cu and Nafion/Cu.........50
Supplementary Fig. 38. Influence of inlet CO_2 humidification on CO_2R performance with a MEA.........51
Supplementary Fig. 39. EIS analysis.........52
Supplementary Fig. 40. Fitted resistances from Nyquist plots for polymer/Cu GDEs.........53
Supplementary Fig. 41. Simulated current density distribution and K^+ diffusion analysis.........54
Supplementary Fig. 42. Products distribution of PT/Cu with different PT loadings..........55
Supplementary Fig. 43. FE_{C2+} on the function of PT loading.........56
Supplementary Fig. 44. FETEM images with varied PT polymer loading.........57
Supplementary Fig. 45. Solution resistance of PT/Cu with varied PT polymer loading.........58
Supplementary Fig. 46. Products distribution in acid electrolyte.........59
Supplementary Fig. 47. SPCE of CO_2R products on PT/Cu electrode at different CO_2 gas flow rate at a current density of −1 A cm^{-2}.........60
Supplementary Fig. 48. Scheme of membrane electrode assembly.........61
Supplementary Fig. 49. MEA performances for PT/Cu.........62
Supplementary Fig. 50. PT coating for MEA reactor and comparison with other works.........63
Supplementary Fig. 51. CO_2R EE versus full cell voltage in PT/Cu based MEA. Electrolyte: 1 M KOH.........64
Supplementary Fig. 52. MEA performance for PT/Cu with 5 M KOH as the electrolyte.........65

Supplementary Fig. 53. CO_2R performance in acid electrolyte with a MEA electrolyser.. 66
Supplementary Fig. 54. CO_2R performance comparison in acid electrolyte with a MEA. 67
Supplementary Tables 68
Supplementary Table 1. The modeling parameters with their associated values. 68
Supplementary Table 2. Rate parameters for charge transfer reactions. 70
Supplementary Table 3. Structure parameters of the catalyst layer and polymer layers. 71
Supplementary Table 4. Water uptake of different polymers immersed in liquid water at 25 °C. 72
Supplementary Table 5. Calculated water uptake capacity of different polymer membranes. 73
Supplementary Table 6. Polymer loading calculation. 74
Supplementary Table 7. CO_2R electrochemical data for PT/Cu with flow cell in 2 M KOH.. 75
Supplementary Table 8. CO_2R electrochemical data for PCR/Cu with flow cell in 2 M KOH.. 76
Supplementary Table 9. CO_2R electrochemical data for PT95/Cu with flow cell in 2 M KOH. 77
Supplementary Table 10. CO_2R electrochemical data for PVDF/Cu with flow cell in 2 M KOH. 78
Supplementary Table 11. CO_2R electrochemical data for Nafion/Cu with flow cell in 2 M KOH.. 79
Supplementary Table 12. CO_2R electrochemical data for the plots in Fig. 5b-f. 80
Supplementary Table 13. CO_2 partial pressure experiment for PT/Cu in Figure 6c and Supplementary Fig. 37. 81

Supplementary Table 14. CO_2 partial pressure experiment for Nafion/Cu in Figure 6c and Supplementary Fig. 37. 82
Supplementary Table 15. EIS fitting resistances for polymer/Cu at varied potentials for the plots of supplementary Fig. 39a..83
Supplementary Table 16. Varied polymer loading and its thickness. 84
Supplementary Table 17. CO_2R electrochemical data for the PT/Cu with varied PT loadings in the plots of Supplementary 42. 85
Supplementary Table 18. CO_2R electrochemical data in acid electrolyte for the plots in Supplementary Fig. 46c. 86
Supplementary Table 19. CO_2R electrochemical data in MEA for the plots in Figure 7a-b and Supplementary Fig. 49...87
Supplementary Table 20. The summary of detailed CO_2R results with MEA in Supplementary Fig. 50c. 88
Supplementary Table 21. Adapted CO_2R electrochemical data for the plots in Figure 7c. 89
Supplementary Table 22. Adapted CO_2R electrochemical data for the plots in Figure 7d. 90
References 91

Supplementary Figures

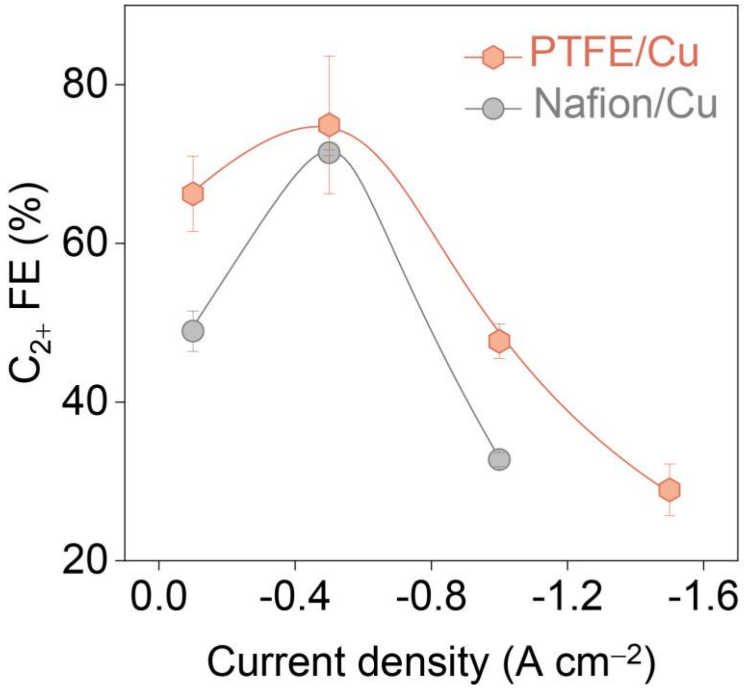

Supplementary Fig. 1. Performance comparison of PTFE/Cu and Nafion/Cu at the same current densities. The error bars represent standard deviations from at least three independent measurements.

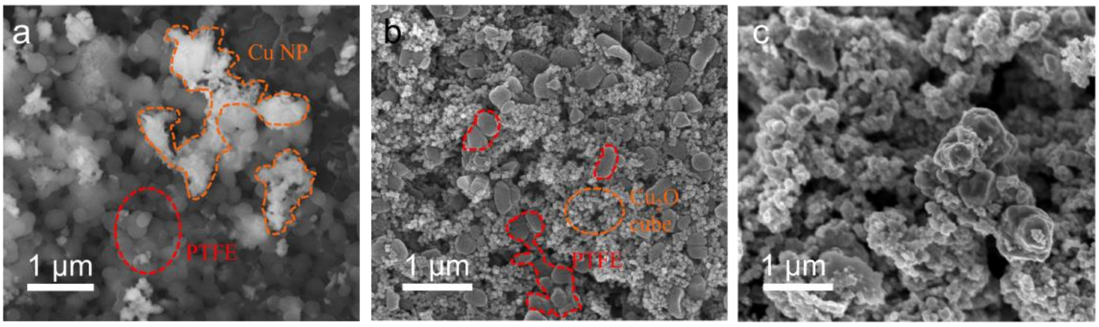

Supplementary Fig. 2. SEM images of polymer/Cu. **a**, PTFE/Cu and **b**, PTFE/Cu_2O cube and **c**, Nafion/Cu. Figure b was used to better show the PTFE particle. To take a good image, Pt/Pd was coated before the SEM analysis to increase the sample conductivity, the coating process was conducted under the current of 20 mA for 40 s. It is noted that no Nafion particles were observed in panel c.

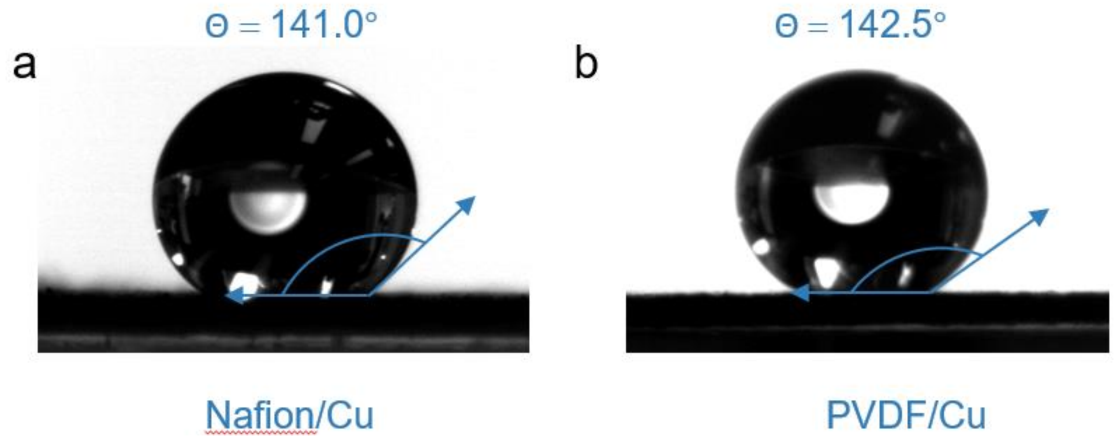

Supplementary Fig. 3. **Contact angles polymer/Cu**. **a**, Nafion/Cu and **b**, PVDF/Cu on the carbon paper

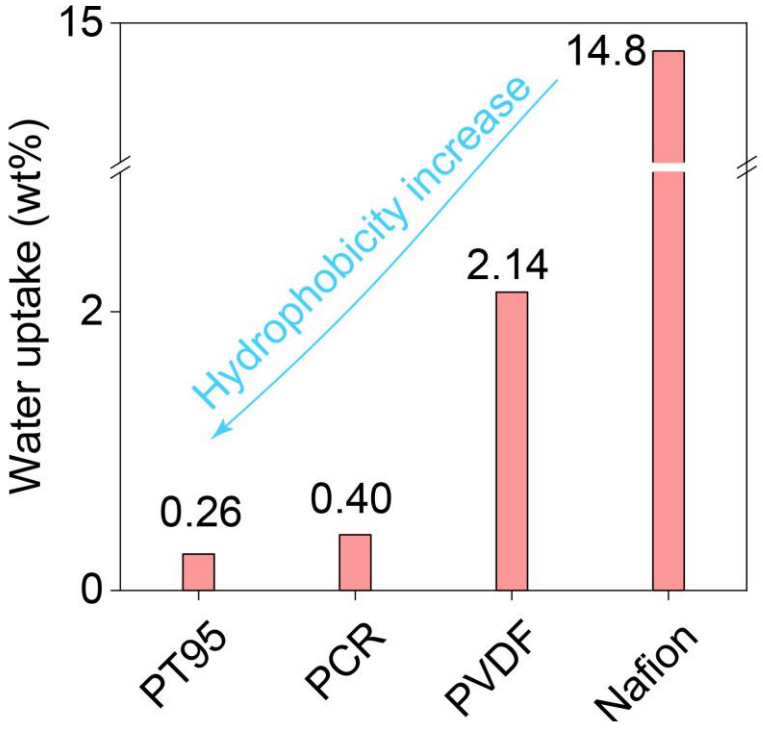

Supplementary Fig. 4. Water uptake ability comparison of polymer membranes.

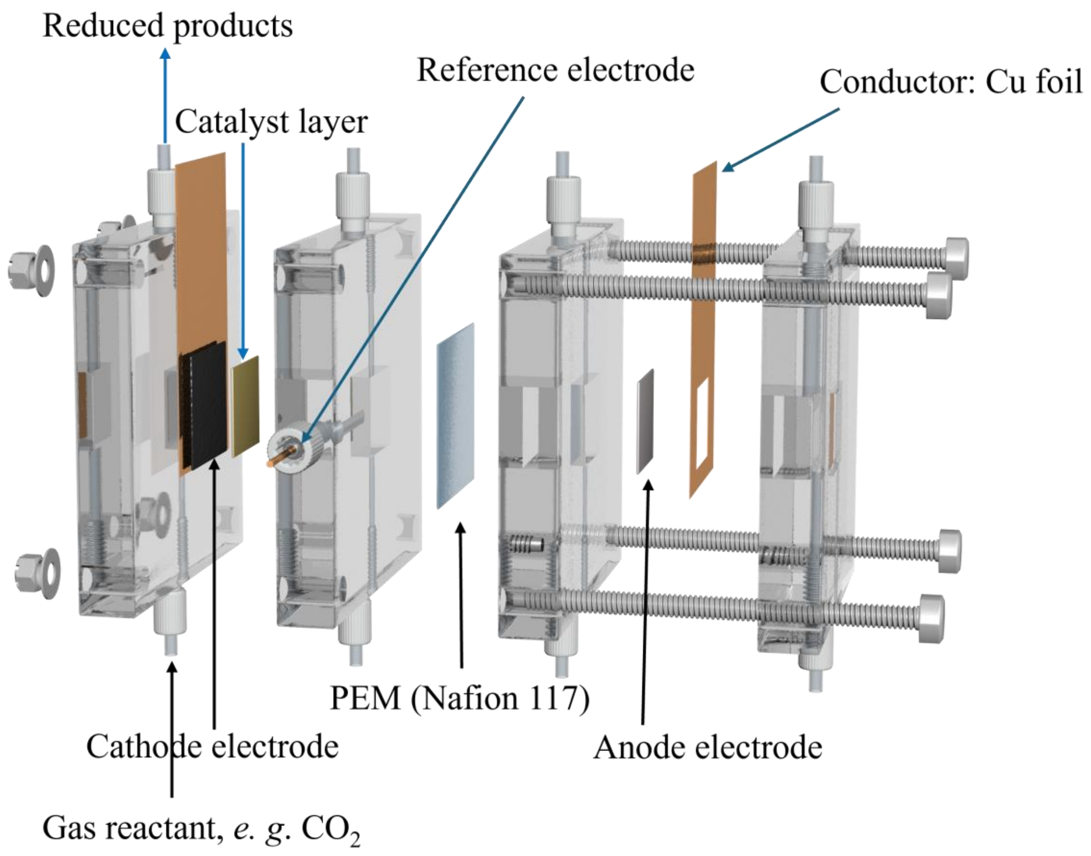

Supplementary Fig. 5. Flow-cell schematic. Reactant gas is fed through the back of a gas diffusion-electrode.

COMSOL simulation

Physical model

In this study, we discuss the performance Cu-Polymers deduced from simulations conducted with a direct pore-level (not volume-averaged) model based on the previous works[1,2]. Fig. 3a and Supplementary Fig. 6 present a schematic representation of the GDE architecture. Assuming that the radius of spherical Cu nanoparticles with a r_{np}, which are loosely packed and covered by a polymer layer (PL)[1], the catalyst layer (CL) is intrinsically porous with a porosity of ε_{CL}. The space among spherical Cu nanoparticles is filled with liquid electrolyte. CO_2 gas either diffuses through this electrolyte region within the pores or migrates via the unsaturated polymer layer (PL) to reach the Cu surfaces[3]. In this 2D simulation, the domain of interest is outlined by red lines, representing a symmetrically reduced region achieved through the application of symmetrical boundary conditions. Methodological development was guided by a catalyst layer modeled with a fixed thickness (Thk_{CL}) derived from the cross-sectional SEM image of Cu-polymer GDEs. The porous polymer layer was employed between the catalyst surface and the liquid electrolyte domain. The polymer thickness is defined as Thk_{PL}, and the mean pore diameter of these three polymers (d_{PL}) is assumed to be the same. To economize computational resources, the liquid electrolyte domain is conceived to be half-sized at 100 nm, complemented with symmetrical boundary conditions. Transport and electrochemical kinetics are resolved in this model to study the CO_2R behavior under different properties of the polymer coating, *i.e.*, thickness, porosity, and ability in managing the water/gas balance at the catalyst surface. The cell performance expressed in terms of product distribution and polarization characteristics was investigated. We explored how catalyst performance toward CO_2 electroreduction would be modified as the polymer layer (PL) varied near the Cu surface. A higher-dimensional model is not necessary because the transport along the out-of-plane thickness direction is relatively slow compared to the in-plane transport[4,5].

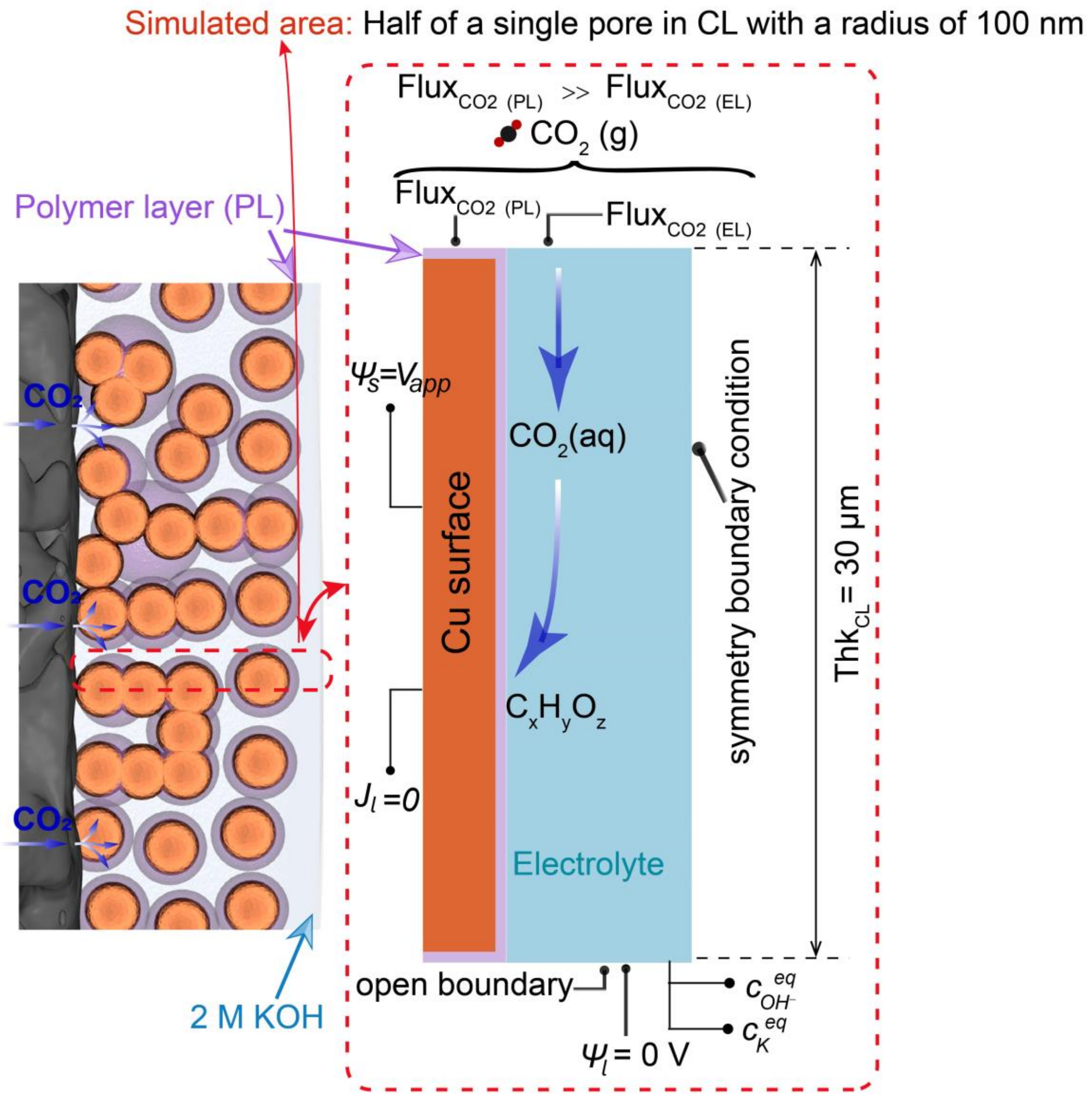

Supplementary Fig. 6. Schematic of modeling framework and boundary conditions. A fixed electrode potential V_{app} was imposed at the left boundary, which contained a Cu cathode with the hydrogen evolution reaction (HER) and CO_2R reactions occur simultaneously. The following cathodic reactions are considered,

$$2H_2O + 2e^- \rightarrow H_2 + 2OH^- \tag{1}$$

$$CO_2 + H_2O + 2e^- \rightarrow CO + 2OH^- \tag{2}$$

$$CO_2 + H_2O + 2e^- \rightarrow HCOO^- + OH^- \tag{3}$$

$$2CO_2 + 8H_2O + 12e^- \rightarrow C_2H_4 + 12OH^- \tag{4}$$

$$2CO_2 + 9H_2O + 12e^- \rightarrow C_2H_5OH + 12OH^- \tag{5}$$

$$3CO_2 + 13H_2O + 18e^- \rightarrow C_3H_7OH + 18OH^- \tag{6}$$

At the gas-electrolyte interface on the top boundary, specific mass-flux conditions for CO_2 mass transfer (Flux $_{CO_2\,(EL)}$ and Flux $_{CO_2\,(PL)}$) were implemented, calculated as follows:

$$\text{Flux}_{\,CO_2\,(EL)} = K_{MT,CO_2\,(EL)}\left(c^0_{CO_2} - c_{CO_2}\right) \tag{7}$$

$$\text{Flux}_{\,CO_2\,(PL)} = K_{MT,CO_2\,(PL)}\left(c^0_{CO_2} - c_{CO_2}\right) \tag{8}$$

The $c^0_{CO_2}$ was specified according to Henry's Law and the Séchenov effect, with zero flux imposed for other charged ions[1]. The gas-to-liquid mass-transfer coefficient, $K_{MT,CO_2\,(EL)}$ and $K_{MT,CO_2\,(PL)}$, are dependent on the thickness of the catalyst layer[3], Thk_{CL}, and the effective CO_2 diffusivity, $D^{eff}_{CO_2\,(EL)}$ and $D^{eff}_{CO_2\,(PL)}$:

$$K_{MT,CO_2\,(EL)} = \frac{D^{eff}_{CO_2\,(EL)}}{Thk_{CL}} \tag{9}$$

$$K_{MT,CO_2\,(PL)} = \frac{D^{eff}_{CO_2\,(PL)}}{Thk_{CL}} \tag{10}$$

A symmetry condition was imposed at the right boundary to model a confined pore geometry in CL. 2 M KOH was employed as the electrolyte in a typical CO_2R measurement. Accordingly, an open boundary condition was imposed at the right boundary, and equilibrium concentration values of K^+ ($c^{eq}_{K^+}$) and OH^- ($c^{eq}_{OH^-}$) were set at this side based on a 2 M KOH electrolyte. Additionally, the electrolyte potential at this boundary was fixed at 0 V, serving as a reference for the electric field calculations. The concentrations of different species including CO_2, HCO_3^-, CO_3^{2-}, H^+, OH^-, H_2O and K^+ were calculated based on Dilute-solution theory, and the water concentration is assumed to be constant. The use of concentrated-solution theory requires additional diffusion coefficients, which may not be easily accessible. However, even with the corrected parameters, it is anticipated that the general trends obtained using dilute-solution theory will remain largely unchanged[3]. The PL domain, which consists of gas, liquid, and solid[6,7], can be viewed as an unsaturated porous medium.

Governing equations

Transport of species in the EL and PL

Mass conservation must be satisfied in the transport of species through the electrolyte layer and polymer layer.

$$\frac{\partial c_i}{\partial t} + \nabla N_i = R_i \tag{11}$$

The molar flux of species i, denoted as N_i, and the volumetric rate of formation of species i, denoted as R_i, must satisfy mass conservation in the transport process through the EL and PL. The rate of production of species i, R_i, can be determined using reactions (25)-(29). The molar flux of species can be expressed as the sum of fluxes due to diffusion and migration.

$$N_i = -D_i{}^{eff} \nabla c_i - z_i u_i F c_i \nabla \phi_l \tag{12}$$

Where c_i represents the concentration of the species i (mol/m^3), $D_i{}^{eff}$ is the diffusion coefficient (m^2/s), u_i is the mobility of ion given by the Nernst–Einstein relationship, and ϕ_l is the electrolyte potential.

The electrolyte current density i_l can be obtained from the total ionic flux,

$$i_l = F \sum_i z_i N_i \tag{13}$$

and the assumption of electro-neutrality,

$$\sum_i z_i c_i = 0 \tag{14}$$

The same set of equations (11)-(14) were used to model the boundary layer region, the electrolyte layer, and the polymer layer.

In EL, the effective diffusion coefficients of species i ($D_{i\,(EL)}^{eff}$) is corrected for the catalyst layer porosity (ε_{CL}) using the Bruggeman relationship,

$$D_{i\,(EL)}^{eff} = \varepsilon_{CL}{}^{\frac{3}{2}} D_i \tag{15}$$

In unsaturated, porous polymer layer, CO_2 within the polymer layer exists in both gas and liquid phases, which in turn improves the gas availability near the Cu surface. The thickness and pore size of the PL are in the order of a few nanometers, which is close to the CO_2 or H_2O molecule diameter. Therefore, in this work, CO_2 within the polymer layer is considered to exist in a homogeneous form, rather than explicitly

exist in gas and liquid phases. In conclusion, it is assumed that the effective CO_2 mass transfer coefficient in this region can be modified by the gas phase CO_2 mass transfer coefficient ($D_{CO_2,g}$) as follows:

$$D_{i\,(PL)}^{eff} = \frac{\theta_l}{\tau_l} D_{i\,(EL)}^{eff} + \frac{\theta_g}{\tau_g} D_{i,g}^{eff} \tag{16}$$

$$\theta_l = \varepsilon_j^{PL} S_j^{PL} \tag{17}$$

$$\theta_g = \varepsilon_j^{PL}\left(1 - S_j^{PL}\right) \tag{18}$$

As for i= HCO_3^-, CO_3^{2-}, H^+, OH^-, and K^+, ${D_{i,g}}^{eff} = 0\ m^2/s$. The volume fraction of H_2O in a pore is defined as the saturation of the polymer j, S_j^{PL}, and is calculated by：

$$S_j^{PL} = \frac{UPT_j^{PL}\rho_j^{PL}}{100\varepsilon_j^{PL}\rho_{H_2O}} \tag{19}$$

Where UPT_j^{PL} (%) is the water uptake of the polymer layer j and ρ_j^{PL} is the density of the polymer. Furthermore, the H_2O/CO_2 ratio of the polymer layer j is then given by:

$$Ratio_j^{PL} = \frac{S_j^{PL}}{1 - S_j^{PL}} \tag{20}$$

$D_{CO_2}^{PL,g}$ is the effective diffusion coefficient of CO_2 in gaseous phase within porous media under standard conditions, which is obtained by adding the Stefan-Maxwell diffusivity and Knudsen diffusivity together and then correcting the result with the Bruggeman relationship:

$$D_{CO_2,g}^{eff} = (\varepsilon_{CL})^{\frac{3}{2}}\left(\frac{1}{D_{CO_2,g}} + \frac{3}{\sqrt{\frac{8RT}{\pi M}}\,d_{PL}}\right)^{-1} \tag{21}$$

Here $D_{CO_2}^{g}$ is the bulk gas diffusion coefficient, d_{PL} is the average pore diameter of the polymer layer and M is the molecular mass of CO_2. ε_j^{PL} is the porosity of the polymer layer j, In the case of Nafion, the porosity is obtained from the isotherms of water desorption for Nafion membranes[8]. The porosities of the other four polymers were determined through CO_2 BET tests (Supplementary Table 2), employing the Dubinin-

Astakhov models to analyze the data[9]. Although the effective diffusivity of CO_2 through the PL decreases substantially relative to its gaseous diffusivity $D_{CO_2,g}$, it remains higher by approximately 10^2 times higher than D_{CO_2} in electrolyte. This ensures a rapid replenishment of CO_2 reactant at the catalytic interface during CO_2R. For dilute electrolytes with concentrations below 10 mol%, the change in diffusion coefficients with respect to electrolyte concentration is insignificant, so we can consider it negligible[10]. Therefore, we can simplify our analysis by neglecting this variation in these effective diffusion coefficients.

Acid-Base Equilibria.

Assuming CO_2 behaves as an ideal gas, the amount of CO_2 dissolved in a solution is determined by the temperature, pressure, and salinity of the solution. Therefore, the value of $c_{CO_2}^0$ can be described by Henry's Law and Séchenov Equation[11,12] as:

$$\log\left(\frac{K_0 f_{CO_2}}{c_{CO_2}^0}\right) = K_s C_S \tag{22}$$

where

$$K_s = \sum(h_{\text{ion}} + h_G) \tag{23}$$

$$h_G = h_{G,0} + h_T(T - 298.15) \tag{24}$$

Where f_{CO_2} refers to the fugacity of CO_2 in gas phase. Other parameters are listed in Supplementary Table 1.

The reactions involving acid/base carbonate and water dissociation in the electrolyte are considered as kinetic expressions rather than assuming equilibrium.

$$\mathrm{CO_2(aq) + H_2O} \overset{k_1,k_{-1}}{\leftrightarrow} \mathrm{H^+ + HCO_3^-}, \qquad K_1 \tag{25}$$

$$\mathrm{HCO_3^-} \overset{k_2,k_{-2}}{\leftrightarrow} \mathrm{H^+ + CO_3^{2-}}, \qquad K_2 \tag{26}$$

$$\mathrm{CO_2(aq) + OH^-} \overset{k_3,k_{-3}}{\leftrightarrow} \mathrm{HCO_3^-}, \qquad K_3 \tag{27}$$

$$\mathrm{HCO_3^- + OH^-} \overset{k_4,k_{-4}}{\leftrightarrow} \mathrm{H_2O + CO_3^{2-}}, \qquad K_4 \tag{28}$$

$$\mathrm{H_2O} \overset{k_\mathrm{w},k_{-w}}{\leftrightarrow} \mathrm{H^+ + OH^-}, \qquad K_\mathrm{w} \tag{29}$$

Where k_n and k_{-n} are the rate constants for the forward and reverse directions of homogeneous reaction, n and K_n is the equilibrium constant. Here, K_n is calculated from Van't Hoff equation, as given by,

$$K_n = \exp\left(\frac{\Delta S_n}{R}\right)\exp\left(-\frac{\Delta H_n}{RT}\right) \tag{30}$$

using the change of entropy, ΔS_n, and the heat of reaction, ΔH_n, listed in Supplementary Table 1.

Charge transfer reactions on the cathode.

The Butler–Volmer equation describes the relationship between the partial current density for product k and the overpotential[13]:

$$i_k = i_{\mathrm{o},k}\prod_i \left(\frac{c_i}{c_i^{\mathrm{ref}}}\right)^{\gamma_{j,k}} \cdot \exp\left(-\frac{\alpha_{\mathrm{c},k}F}{RT}\eta_k\right) \tag{31}$$

η_k represents the overpotential, which is calculated as the difference between the electrode potential after Nernstian correction and the equilibrium potential,

$$\eta_k = (\phi_{\mathrm{S}} - \phi_{\mathrm{L}}) - \left(U_k^{\mathrm{o}} - \frac{2.303RT}{F}\mathrm{pH}\right) \tag{32}$$

Here, U_k^{o} is the reference potential of a reaction k. In our simulation, we utilized kinetic parameters that were obtained from Adam's[13] experimental measurements on roughened Cu. It should be noted, however, that the kinetics in the CL environment may not be identical to those in the aqueous solution, as they may involve different ion concentrations and types. Nonetheless, in the absence of any alternative data, we employed these kinetic parameters to investigate the operation of the local electrochemical reactions on the Cu nanoparticles surface. These rate parameters for charge transfer reactions are shown in Supplementary Table 2.

Size reduction with x-coordinate transformation.

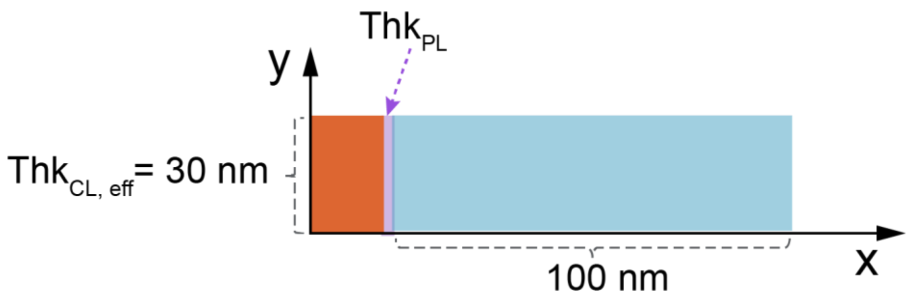

Supplementary Fig. 7. Schematic of the dimensions of each domain in the simulation model.

Due to the catalyst layer thickness (30 μm), which is much larger than the average radius of the catalyst pores (100 nm), achieving convergence in a 2D model presents considerable challenges. Consequently, the presented model uses the x-coordinate (catalyst layer thickness direction) transformation to reduce the size of the domain by a factor of F_R:

$$F_R = \frac{Thk_{CL}}{Thk_{CL,eff}} \tag{33}$$

As shown in Supplementary Fig. 7, $Thk_{CL,eff}$ is the catalyst layer thickness after modification. In this model, isotropic effective properties are employed, with modifications applied only to properties in the x-direction by the factor F_R, since only the x-coordinate is transformed.

$$D_{i,eff,x} = \frac{D_{i,eff}}{F_R} \tag{34}$$

$$\varepsilon_x = \frac{\varepsilon}{F_R} \tag{35}$$

$$R_{i,x} = F_R R_i \tag{36}$$

$$\rho_{,x} = \rho F_R \tag{37}$$

Sophia Haussener et al. have conducted verification that this size reduction transformation was implemented properly[2].

These related structure parameters of the catalyst layer and polymer layers are listed in Supplementary Table 3.

To solve the governing equations, we utilized the COMSOL Multiphysics 5.6 software and employed the PARDISO solver with a relative tolerance of 0.001. The maximum element size in the modeling domain was set to 0.1 nm. To accurately capture sharp concentration gradients, we decreased the element sizes to 0.01 nm near the boundary of cathode surface, PL, and EL. A steady-state study was performed to simulate multi-physics evolution.

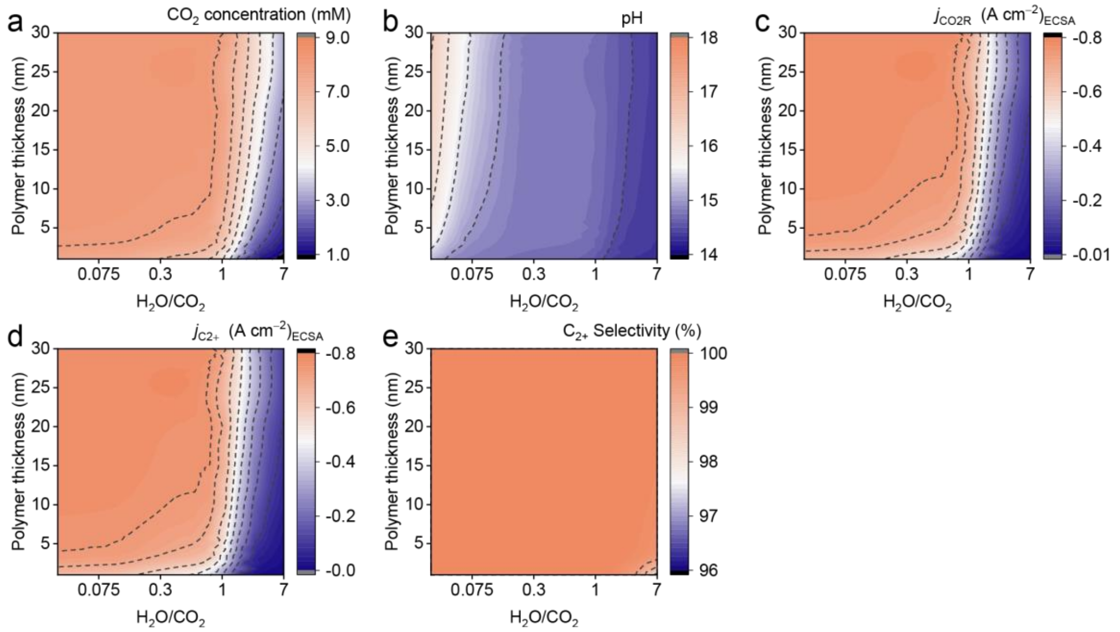

Supplementary Fig. 8. **Modeled local species profile with varied polymer thickness and H_2O/CO_2 ratio**. **a**, local CO_2 concentration, **b**, local pH, **c**, CO_2R current density (exclude H_2), **d**, C_{2+} partial current density and **e**, C_{2+} selectivity with the variation of polymer thickness and H_2O/CO_2 ratio.

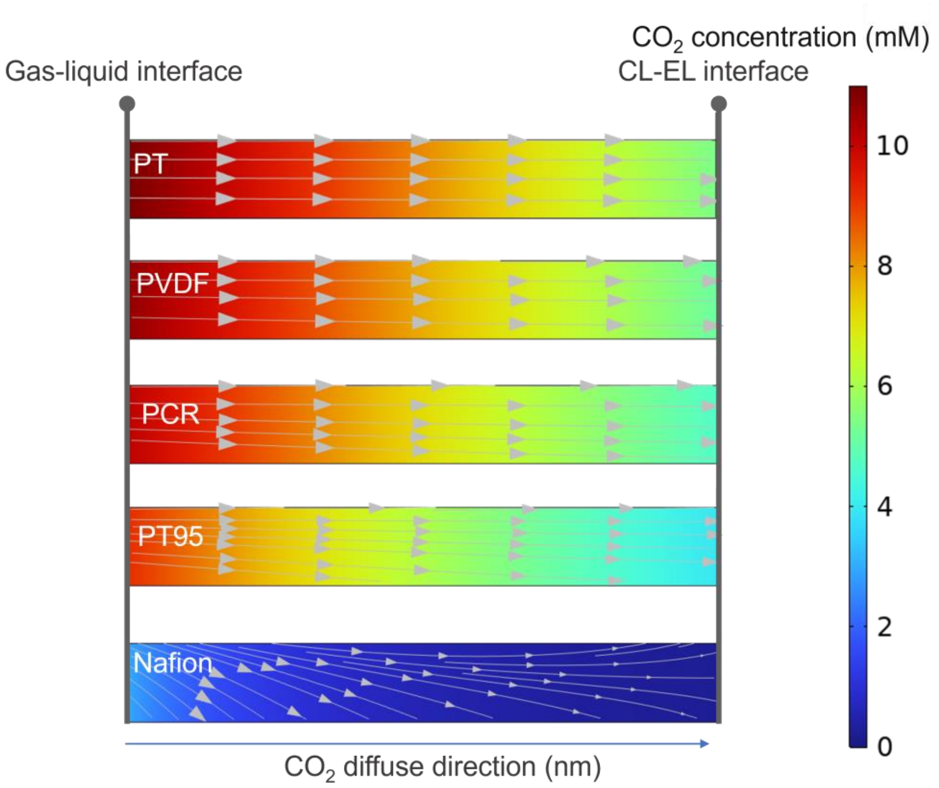

Supplementary Fig. 9. CO_2 concentration distribution and streamline distribution within the polymer layer at −1.426 V vs. SHE.

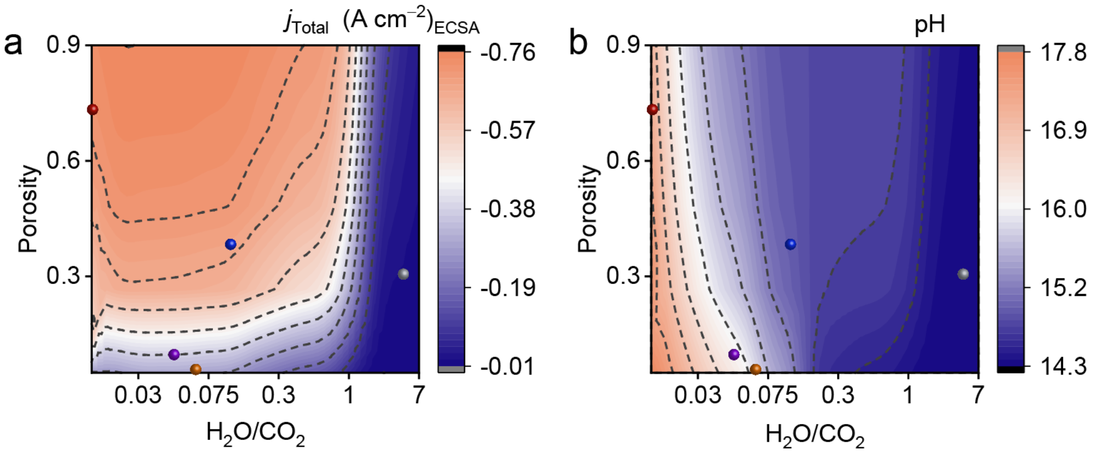

Supplementary Fig. 10. **Modeled local species profile with varied polymer porosity and H_2O/CO_2 ratio**. **a**, CO_2R current density (excluding H_2) and **b**, local pH with the variation of polymer porosity and H_2O/CO_2 ratio. The five spherical symbols in Figures represent the experimentally measured/derived porosity and local H_2O/CO_2 ratio of five polymers used in this study (vide infra). Red: PT; Purple: PCR; Orange: PT95; blue: PVDF; gray: Nafion.

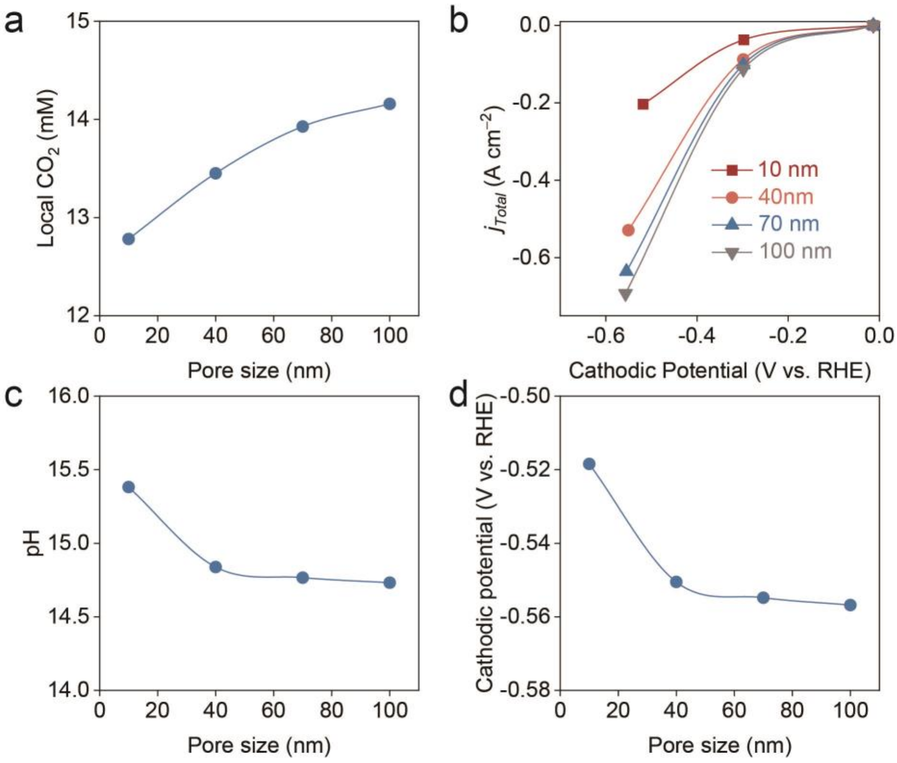

Supplementary Fig. 11. **Influence of the pore size of the catalyst layer**. **a**, Comparison of average local CO_2 concentration, **b**, simulated polarization curves, **c**, average local pH and **d**, cathodic potential versus RHE for GDEs with catalyst layers of varying pore sizes. In all cases, the porosity of the catalyst layer is maintained at 0.5, the H_2O/CO_2 ratio in the polymer is set at 0.1, and the applied potential at the catalyst is fixed at –1.426 V.

Note: We used an air-brushing method to prepare the GDEs. Due to the high evaporation rate of the solvent (*i. e.*, ethanol and FC-770), as well as the micrometer scale and high surface-to-volume ratio aerosol mists (catalyst ink) induced by the pressurized gas, the resulting catalyst layers are porous, with well scattered nanoparticles before they can settle down into the microporous layer. This porous structure has been demonstrated in a previous report[14], where the airbrushed catalyst layer was four times thicker and more porous than the drop-casted and hand-painted layers. Similar porous catalyst structure, achieved through the airbrushing method, was also observed in another report[15]. In this work, an average pore size of approximately 230 nm was determined by Focused Ion Beam (FIB).

We also checked our catalyst layer by the SEM images of these polymer/Cu GDEs, As illustrated in Supplementary Fig. 12, porous structures were observed for all the prepared GDEs, with most measured pore sizes larger than 200 nm in PT/Cu (pores in other polymer/Cu GDEs are even larger). Although achieving even and precise control over the pore size of the catalyst layer is extremely challenging practically, we tried to keep our catalyst layer preparation protocols consistent for all the polymer/Cu

GDEs in this study, to ensure relatively large pore sizes based on ours and documented experiences. Based on these considerations, we set the pore diameters within the catalyst layer in the current simulations to be a constant value of 200 nm to achieve reasonable comparisons. Consequently, the reasonably good match between the simulation results and our experimental observations confirms that the real pore size within the catalyst layers in our studies is substantially larger than 10 nm.

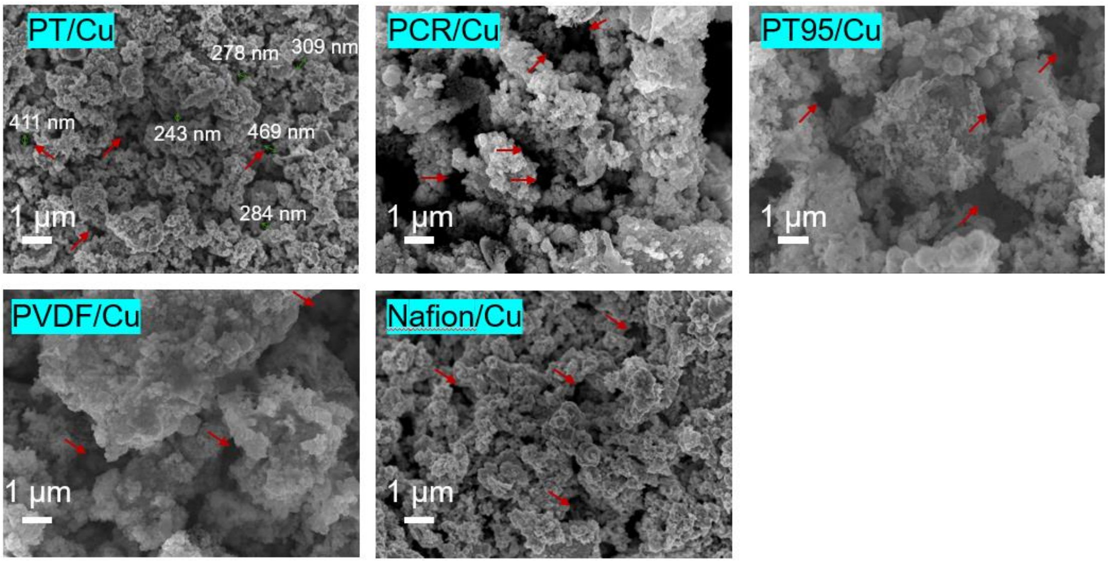

Supplementary Fig. 12. **SEM images of polymer/Cu GDEs**. The red arrows indicate typical pores in the catalyst layer.

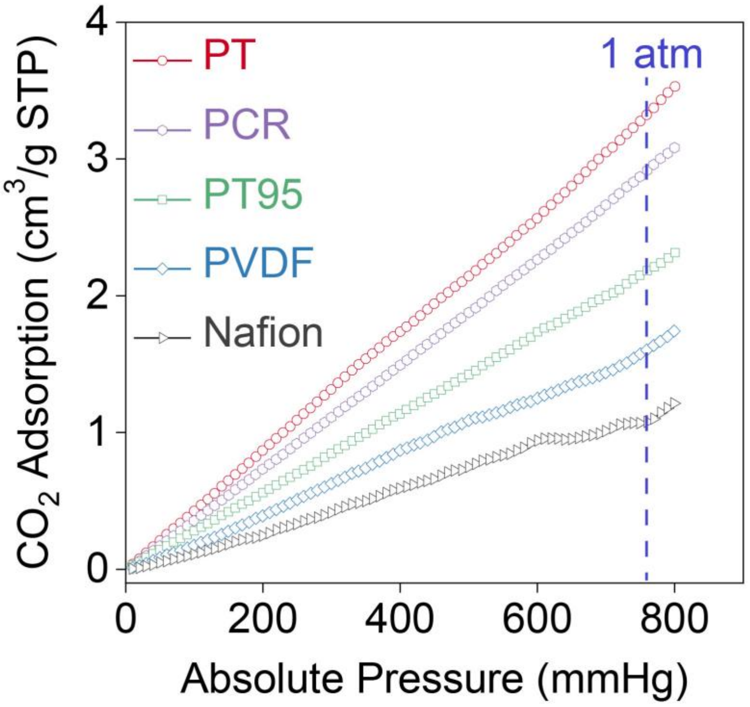

Supplementary Fig. 13. CO_2 adsorption isotherms of polymer membranes under varied pressure.

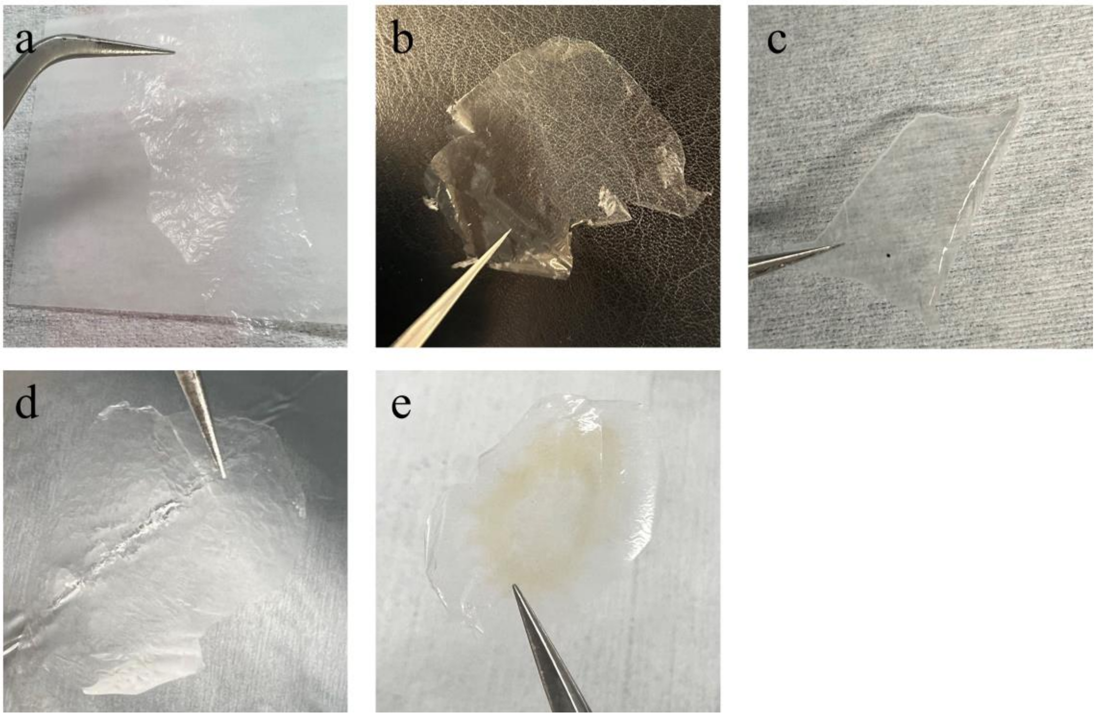

Supplementary Fig. 14. **Photograph of polymer membranes**. **a**, PT, **b**, PCR, **c**, PT95, **d**, PVDF and **e**, Nafion membranes. These polymer membranes were made by a drop casting method.

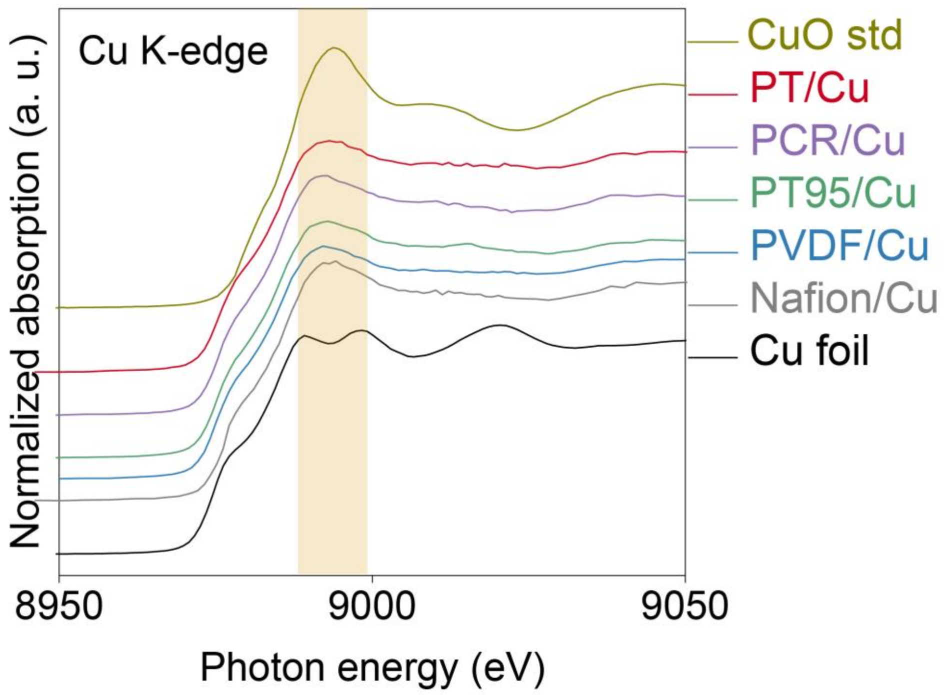

Supplementary Fig. 15. Cu K-edge spectra of the five polymer/Cu catalysts.

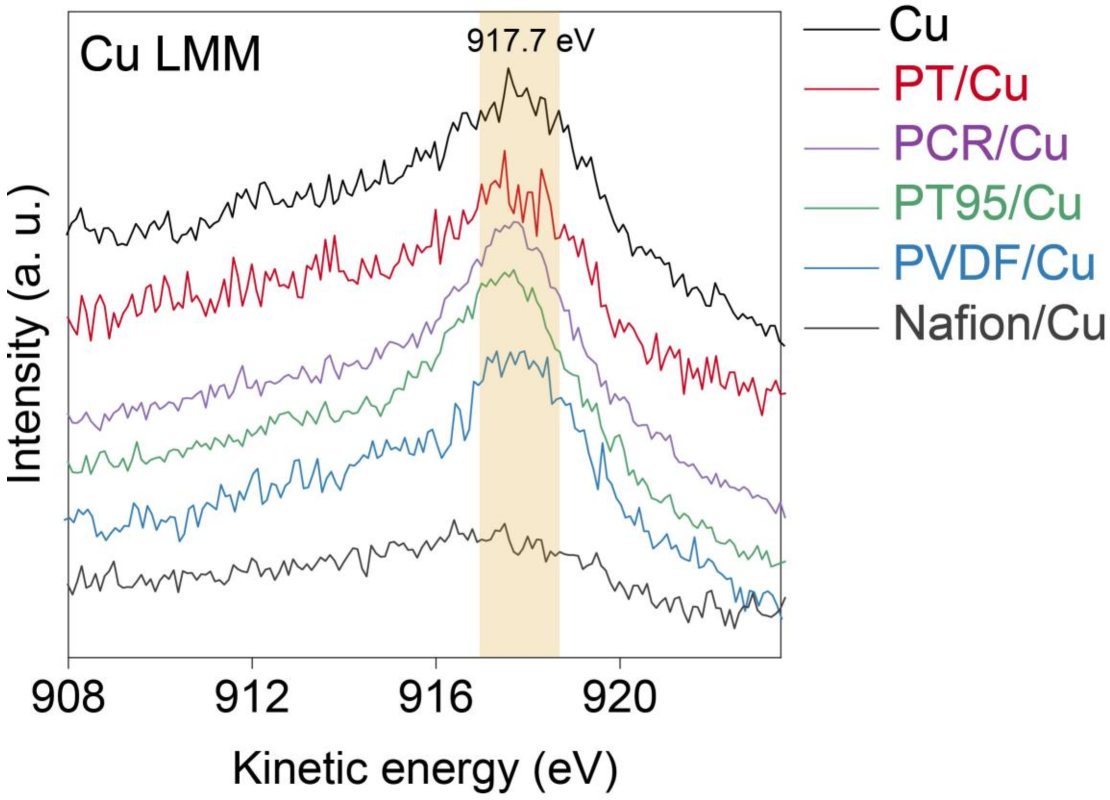

Supplementary Fig. 16. Cu LMM Auger spectra of Cu and polymer/Cu.

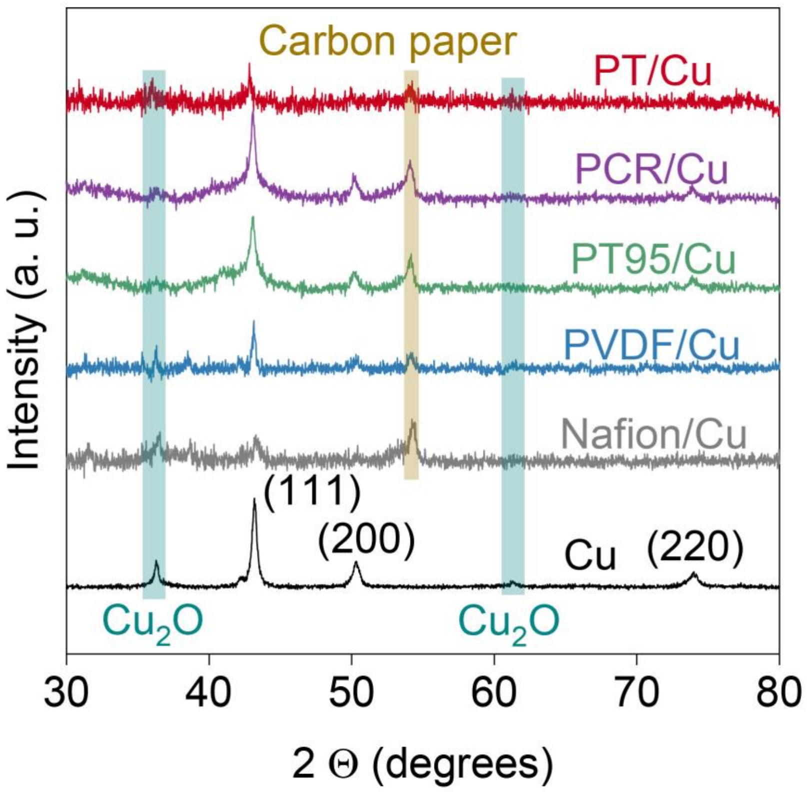

Supplementary Fig. 17. XRD pattern of polymer/Cu and initial Cu.

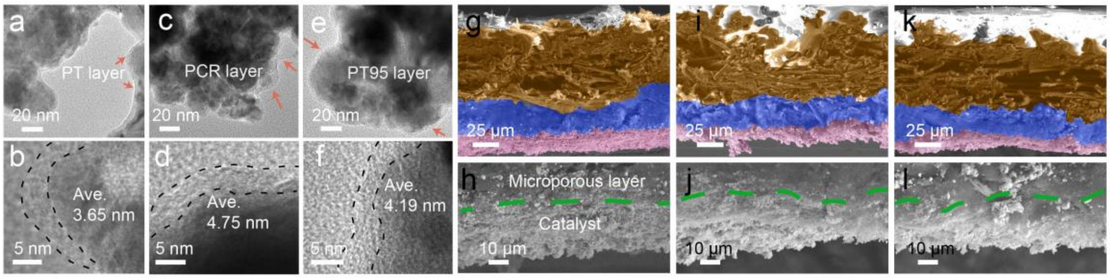

Supplementary Fig. 18. **TEM and cross-section characterizations for PT/Cu, PCR/Cu and PT95/Cu GDEs**. **a-f**, FETEM images of (**a-b**) PT/Cu, (**c-d**) PCR/Cu and (**e-f**) PT95/Cu. **g-l**, cross-sections of (**g-h**) PT/Cu, (**i-j**) PCR/Cu and (**k-l**) PT95/Cu based GDEs. In Figure **g-k**, false colors applied to the images for clarity, pink: catalyst layer, blue: microporous layer, orange: gas diffusion layer. The white color on the top of the GDE is the pre-treated PTFE layer.

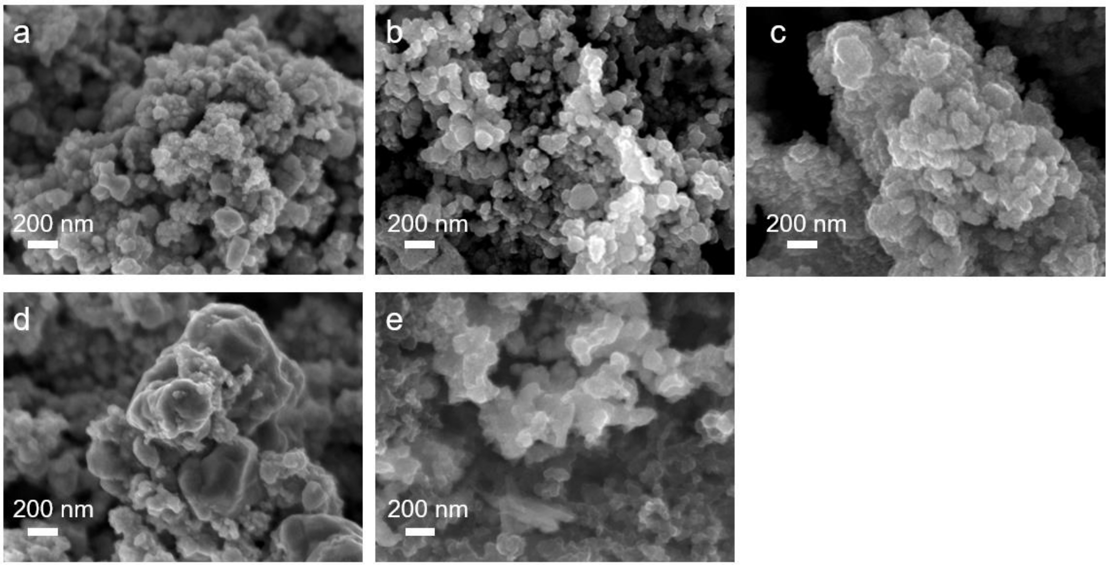

Supplementary Fig. 19. **SEM images of Cu with different polymer coatings**. **a**, PT/Cu, **b**, PT95/Cu, **c**, PCR/Cu, **d**, Nafion/Cu and **e**, PVDF/Cu.

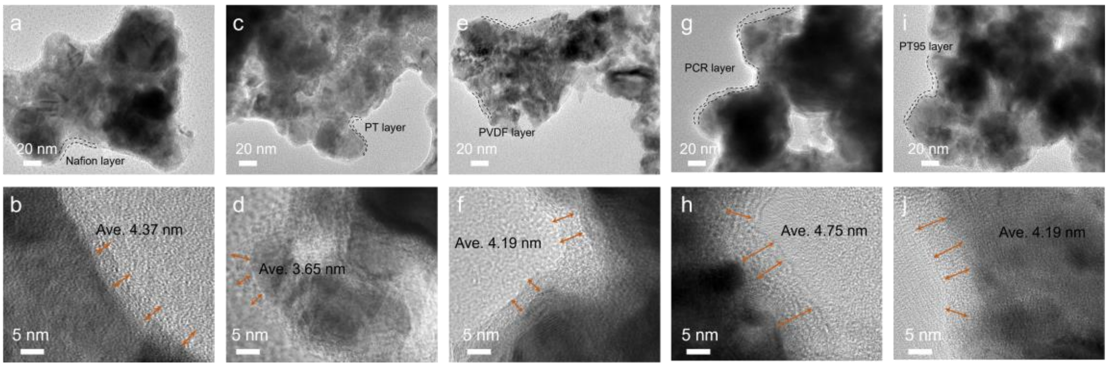

Supplementary Fig. 20. TEM images of Cu NP with different polymer coatings. **a-b**, Nafion/Cu, **c-d**, PT/Cu and **e-f**, PVDF/Cu, **g-h**, PCR/Cu and **i-j**, PT95/Cu.

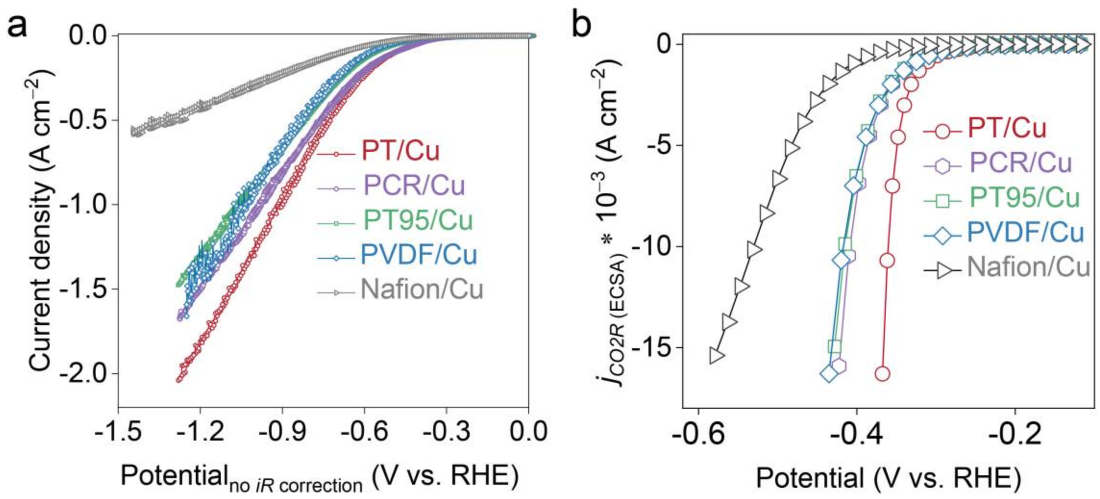

Supplementary Fig. 21. Tested and simulated polarization curves comparison of polymer/Cu GDEs. The solution resistance for PT/Cu, PCR/Cu, PT95/Cu, PVDF/Cu and Nafion/Cu are 0.31 Ω, 0.35 Ω, 0.33 Ω, 0.31 Ω and 0.38 Ω, respectively.

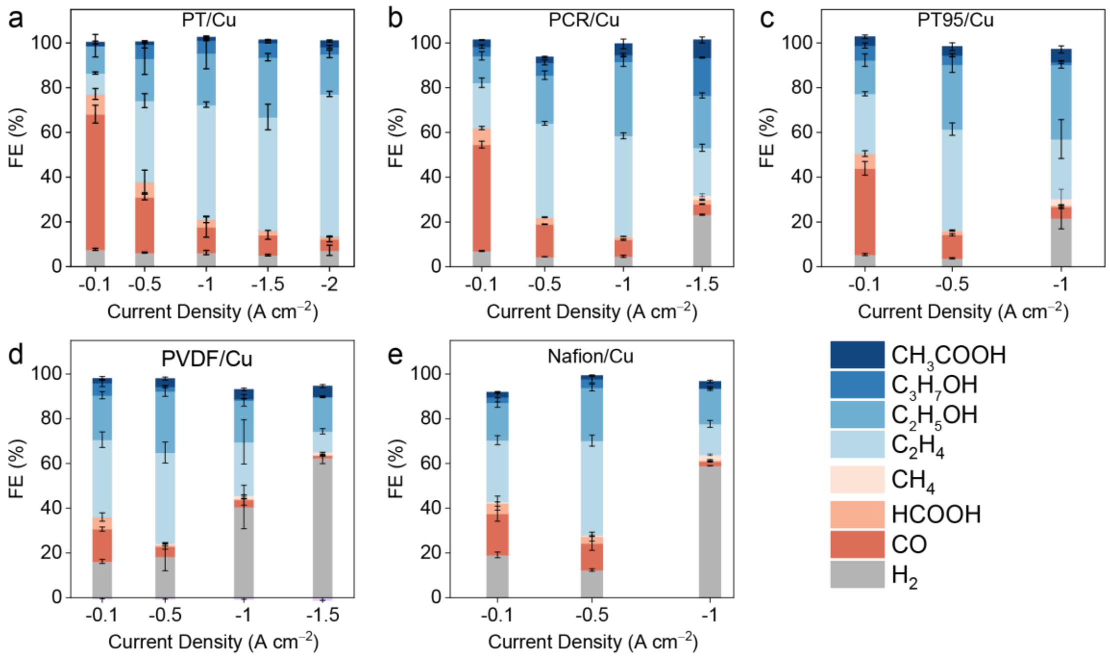

Supplementary Fig. 22. **Faradic efficiencies for CO2R products**. **a**, PT/Cu, **b**, PCR/Cu, c, PT95/Cu, **d**, PVDF/Cu and **e**, Nafion/Cu. The error bars represent standard deviations from at least three independent measurements. Relevant source data are provided as a Source Data file.

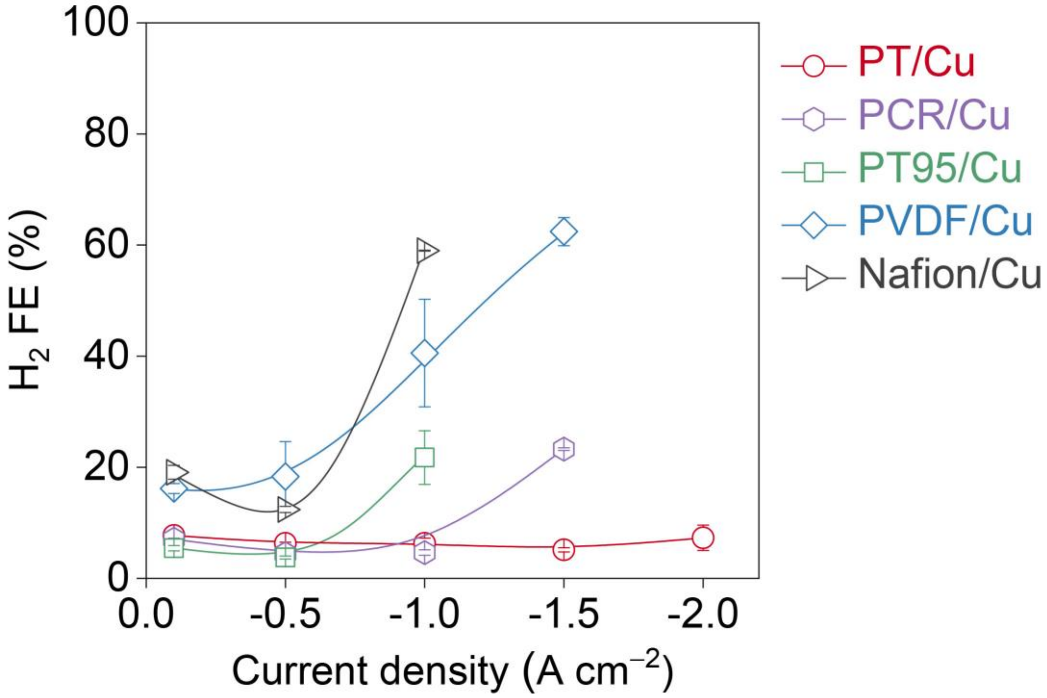

Supplementary Fig. 23. H_2 activity comparison of FE versus current densities. The error bars represent standard deviations from at least three independent measurements.

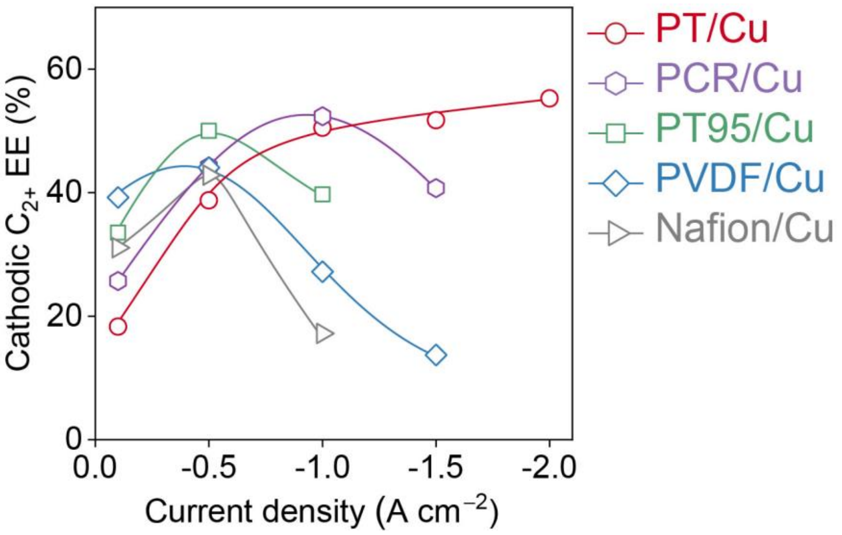

Supplementary Fig. 24. C_{2+} cathodic energy efficiency comparison for polymer/Cu GDEs.

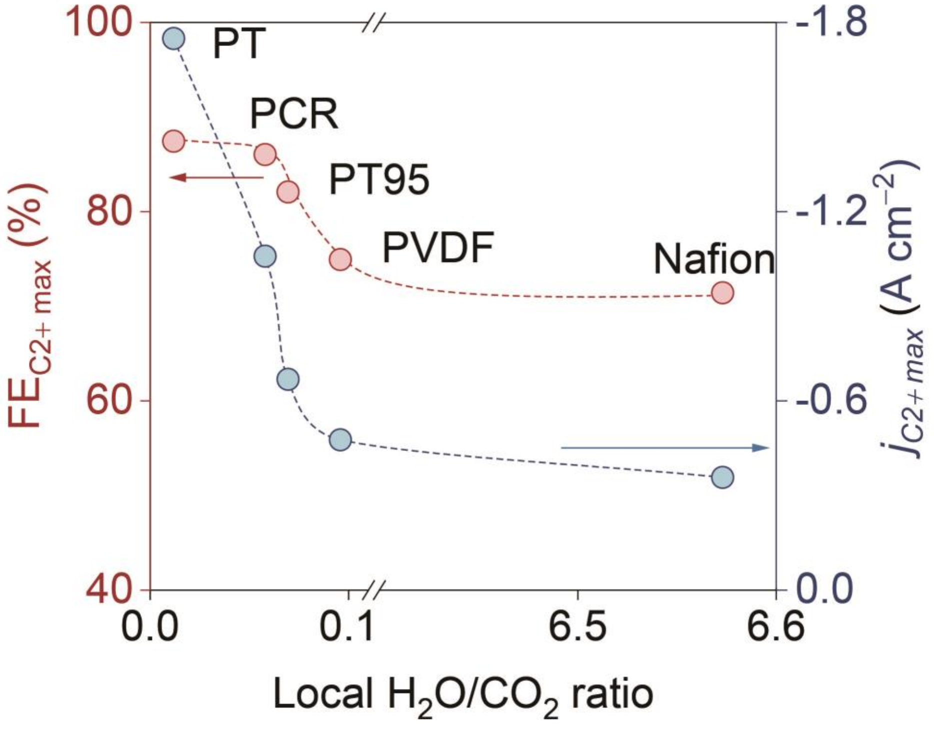

Supplementary Fig. 25. Correlation between regulated local H_2O/CO_2 ratio and the performance of C_{2+} products from CO_2R with five polymer/Cu GDEs.

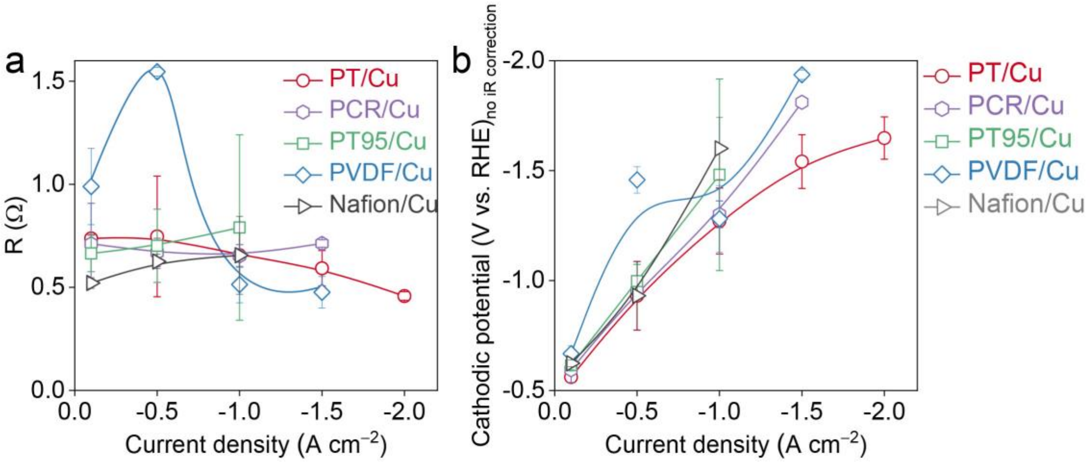

Supplementary Fig. 26. **Cathodic potential comparison for five polymer/Cu GDEs**. **a**, tested R and **b**, cathodic potentials for five polymer/Cu electrodes without *iR* correction under different current densities. The error bars represent standard deviations from at least three independent measurements.

Note: The values of the solution resistances in panel a are shown below:

PT/Cu: 0.74 ±0.02, 0.75±0.29, 0.66±0.14, 0.59±0.09, 0.46±0.02 at current density of –0.1, –0.5, –1, –1.5, –2 A cm^{-2}, respectively.

PCR/Cu: 0.71±0.19, 0.67±0.07, 0.65±0.05, 0.71±0.02 at current density of –0.1, –0.5, –1, –1.5 A cm^{-2}, respectively.

PT95/Cu: 0.66±0.09, 0.70±0.18, 0.79±0.45 at current density of –0.1, –0.5, –1 A cm^{-2}, respectively.

PVDF/Cu: 0.99±0.19, 1.55±0.02, 0.51±0.09, 0.48±0.08 at current density of –0.1, –0.5, –1 A cm^{-2}, respectively.

Nafion/Cu: 0.52±0.03, 0.63±0.03, 0.66±0.19 at current density of –0.1, –0.5, –1 A cm^{-2}, respectively.

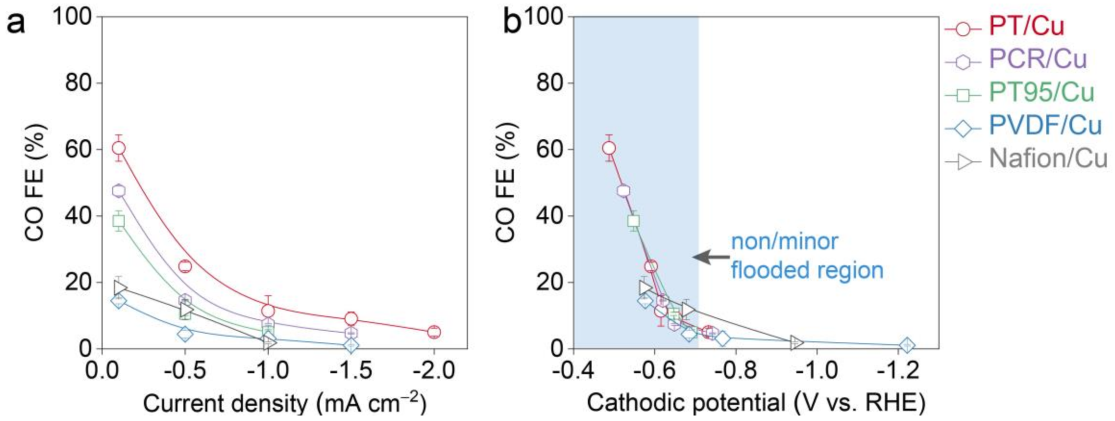

Supplementary Fig. 27. **CO FE comparison for five polymer/Cu GDEs**. Comparison at different **a**, current densities and **b**, cathodic potentials. The error bars represent standard deviations from at least three independent measurements.

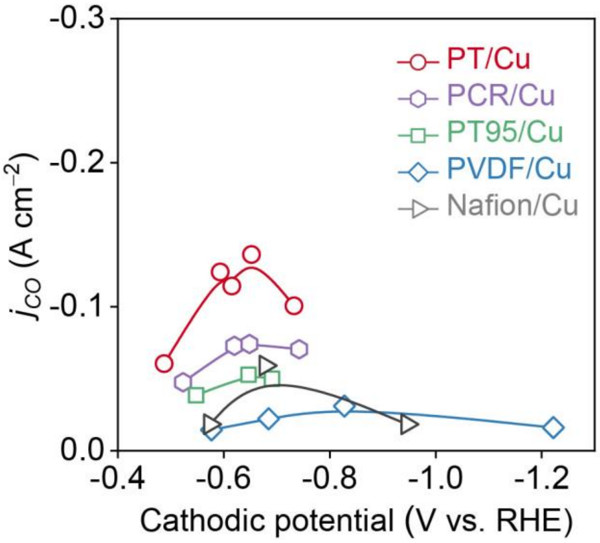

Supplementary Fig. 28. CO partial current density comparison at the same cathodic potentials.

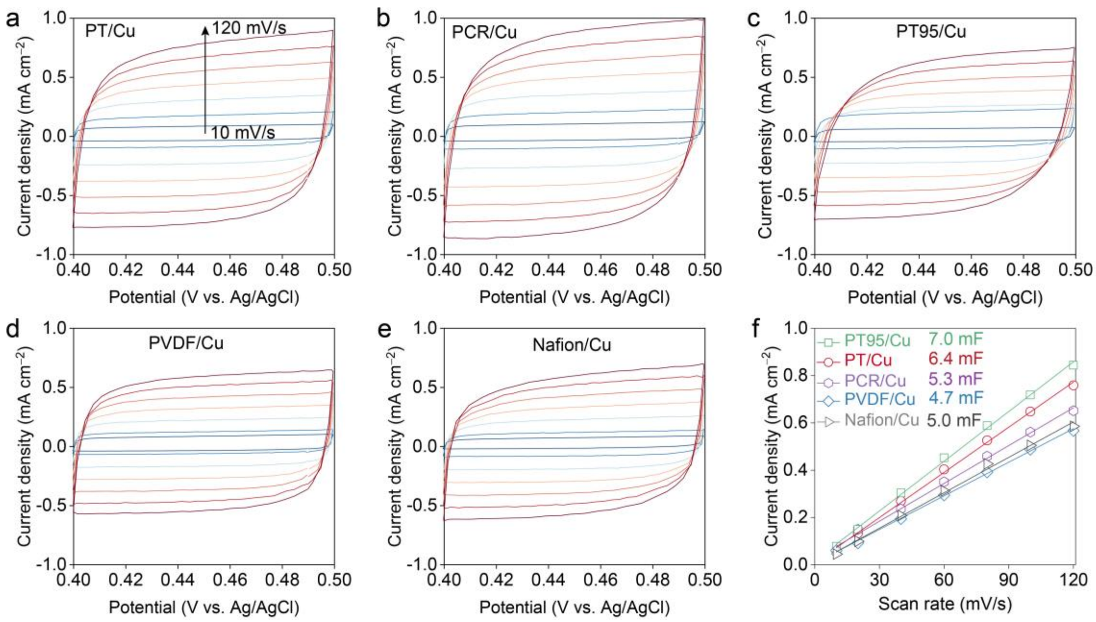

Supplementary Fig. 29. **ECSA comparison**. Cyclic voltammetry curves for **a**, PT/Cu, **b**, PCR/Cu, **c**, PT95/Cu, **d**, PVDF/Cu and **e**, Nafion/Cu with the scan rate from 10 mV s^{-1} to 120 mV s^{-1}, and **f**, double layer capacitance for all five polymer/Cu electrodes. ECSA testing was carried out in an H-Cell. The polymer/Cu catalysts were spray-coated onto carbon paper (P75T) with a $1*1$ cm^2 area, achieving a Cu loading of ~1 mg cm^{-2}.

Note: To derive panel f, from the CV curves, plot the current density (j) at the middle of the potential window (0.45 V vs. Ag/AgCl) versus the scan rate (mV/s), the slope of f gives the double-layer capacitance. Normally, ECSA is calculated using the formula: $ECSA = \frac{C_{dl}}{C_s}$, where C_{dl} is the measured double-layer capacitance and C_s is the specific capacitance. As the specific capacitance is unknown, we assume it is the same for all the electrodes. Thus, with higher double-layer capacitance should possess higher ECSA values.

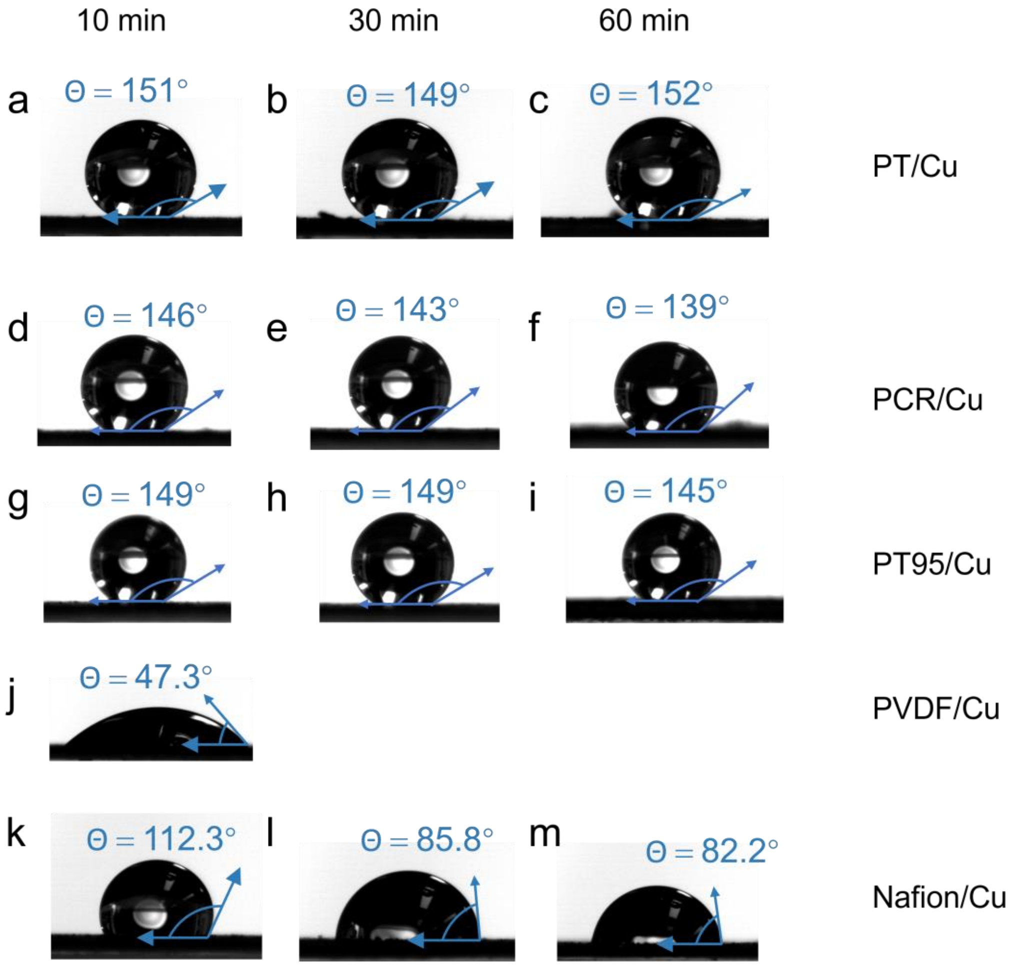

Supplementary Fig. 30. **Post contact angles**. **a-c**, 10 min, 30 min and 1 h for PT/Cu, **d-f**, 10 min, 30 min and 1 h for PCR/Cu, **g-i**, 10 min, 30 min and 1 h for PT95/Cu, **j**, 10 min for PVDF/Cu and **k-m**, 10 min, 30 min and 1 h for Nafion/Cu. All tests were at current density of –0.5 A cm^{-2}.

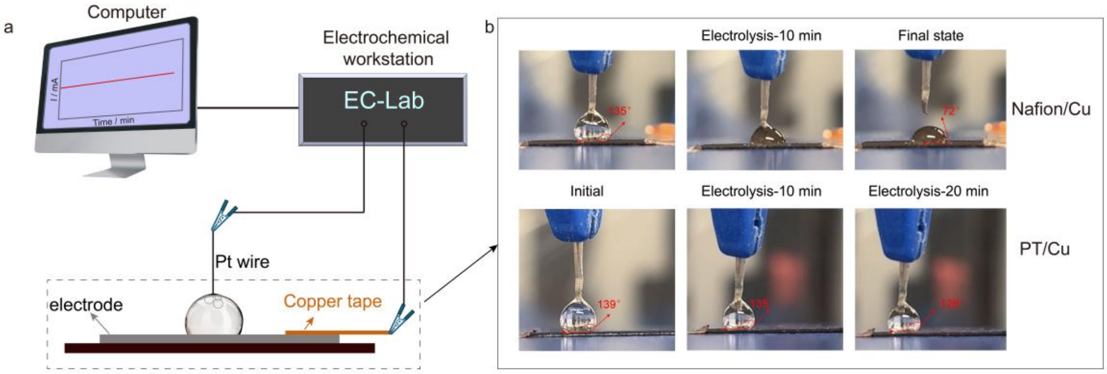

Supplementary Fig. 31. **Visualization of the electrowetting effect**. **a**, Schematic of the custom-made device for *in-situ* contact angle test. **b**, the contact angle change of the electrolyte droplet on the Nafion/Cu and PT/Cu electrode were monitored in real time at a current of 20 mA. The droplet is electrolyte used for CO_2R.

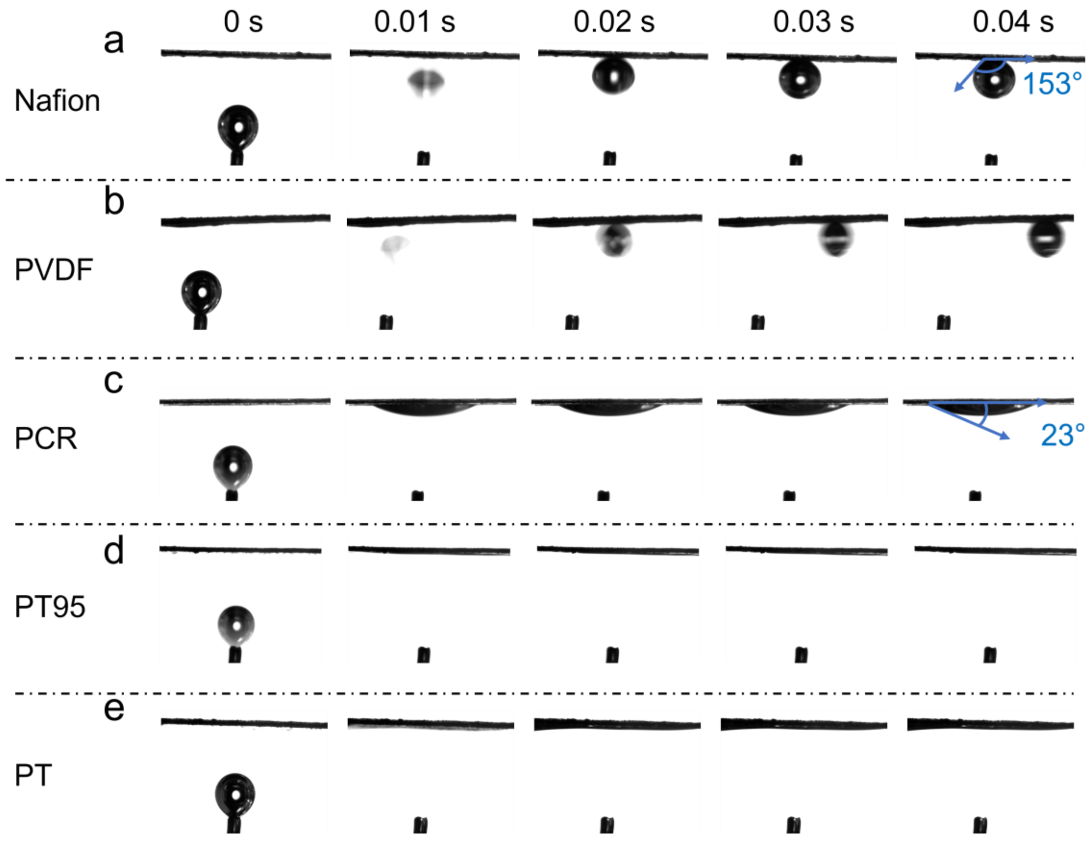

Supplementary Fig. 32. **Post gas bubble adhesion behaviors**. The optical pictures and corresponding air contact angle for **a**, Nafion/Cu, **b**, PVDF/Cu, **c**, PCR/Cu, **d**, PT95/Cu and **e**, PT/Cu using pure water as solution. These electrodes have undergone CO_2R electrolysis for 30 min at –0.5 A cm^{-2}.

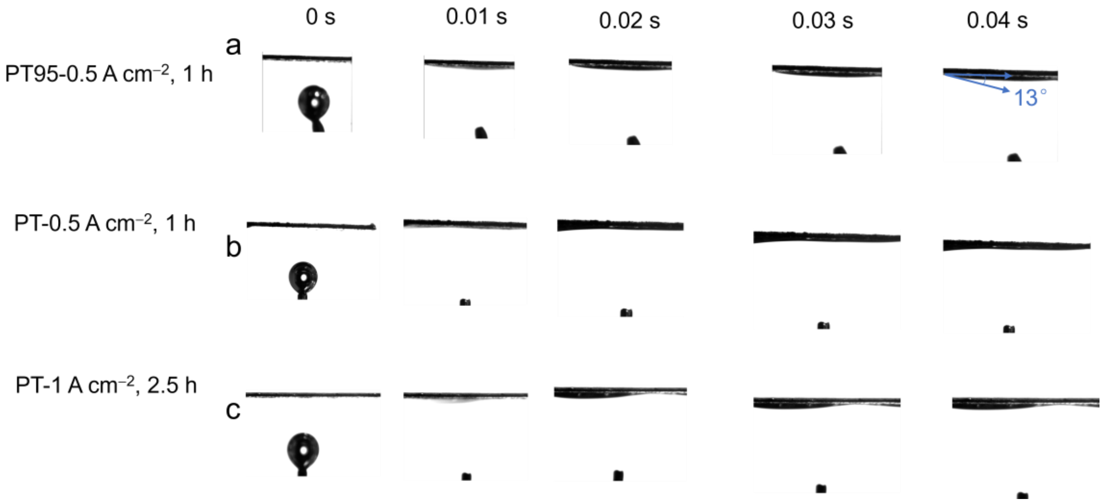

Supplementary Fig. 33. **Post gas bubble adhesion behaviors at extended current density and time**. The optical pictures and corresponding air contact angle for **a**, PT95/Cu, **b**, PT/Cu, the above two electrodes were after electrolysis at –0.5 A cm^{-2} for 1 h; **c**, PT/Cu after electrolysis at –2.5 A cm^{-2} for 2.5 h.

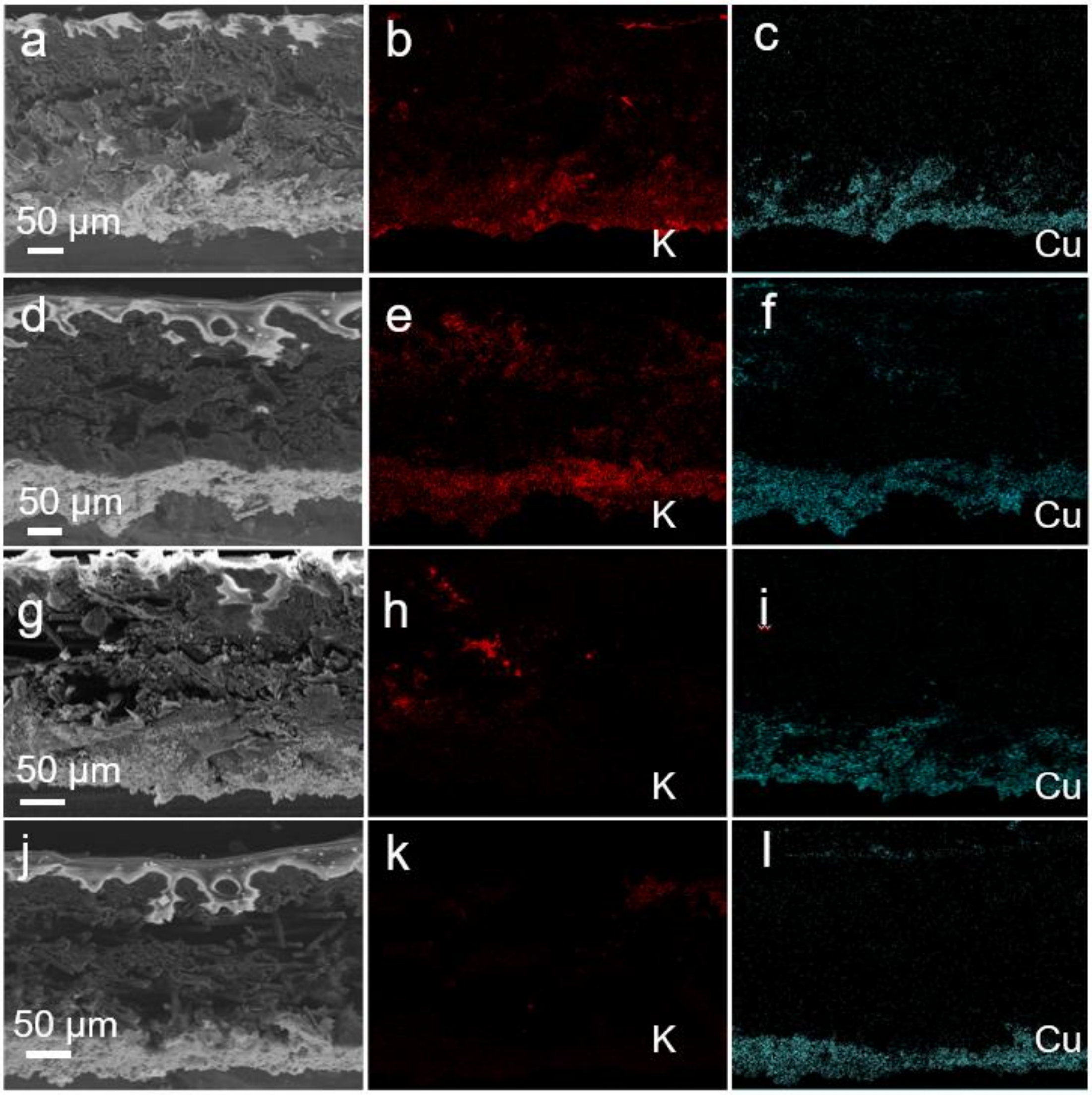

Supplementary Fig. 34. **Post cross-sectional scanning electron microscopy and energy-dispersive X-ray spectroscopy elemental mapping images**. **a-c**, Nafion/Cu, **d-f**, PVDF/Cu GDE and **g-i**, PT/Cu GDE after electrolysis at –0.5 A m^{-2} for 30 min, **j-l**, PT/Cu GDE electrolysis at –1.5 A cm^{-2} for 30 min. The scale bars in the elemental mapping images are the same as those in their corresponding scanning electron microscopy images.

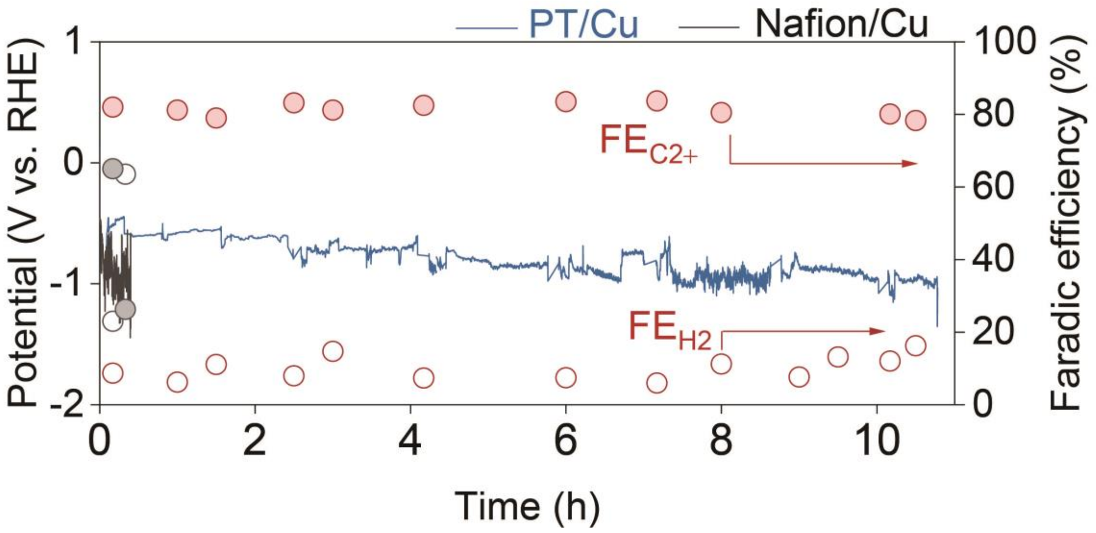

Supplementary Fig. 35. Stability of PT/Cu and Nafion/Cu at −1 A cm^{-2} in 1 M KOH.

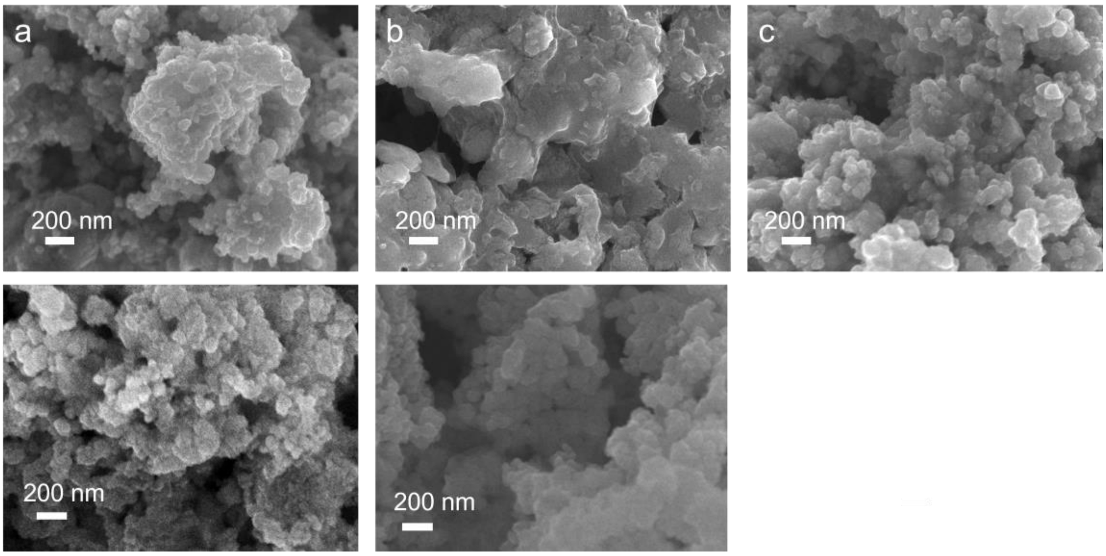

Supplementary Fig. 36. **Post SEM for different polymer/Cu**. **a**, PT/Cu, **b**, PVDF/Cu and **c**, Nafion/Cu, **d**, PCR/Cu and **e**, PT95/Cu. All GDEs were tested at –0.5 A cm^{-2} for 30 min.

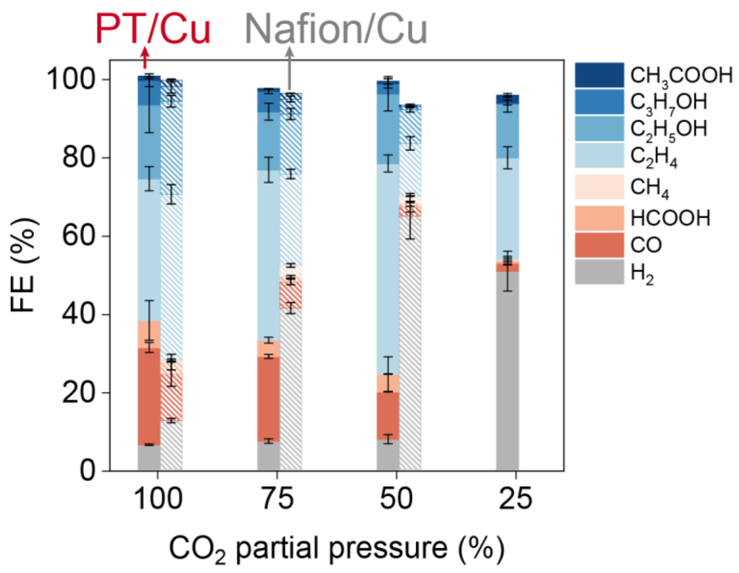

Supplementary Fig. 37. CO_2 partial pressure experiment for PT/Cu and Nafion/Cu. Test conditions: current density: –0.5 A cm^{-2}; electrolyte: 2 M KOH; total gas flow: 24 sccm. The error bars represent standard deviations from at least three independent measurements. Relevant source data are provided as a Source Data file.

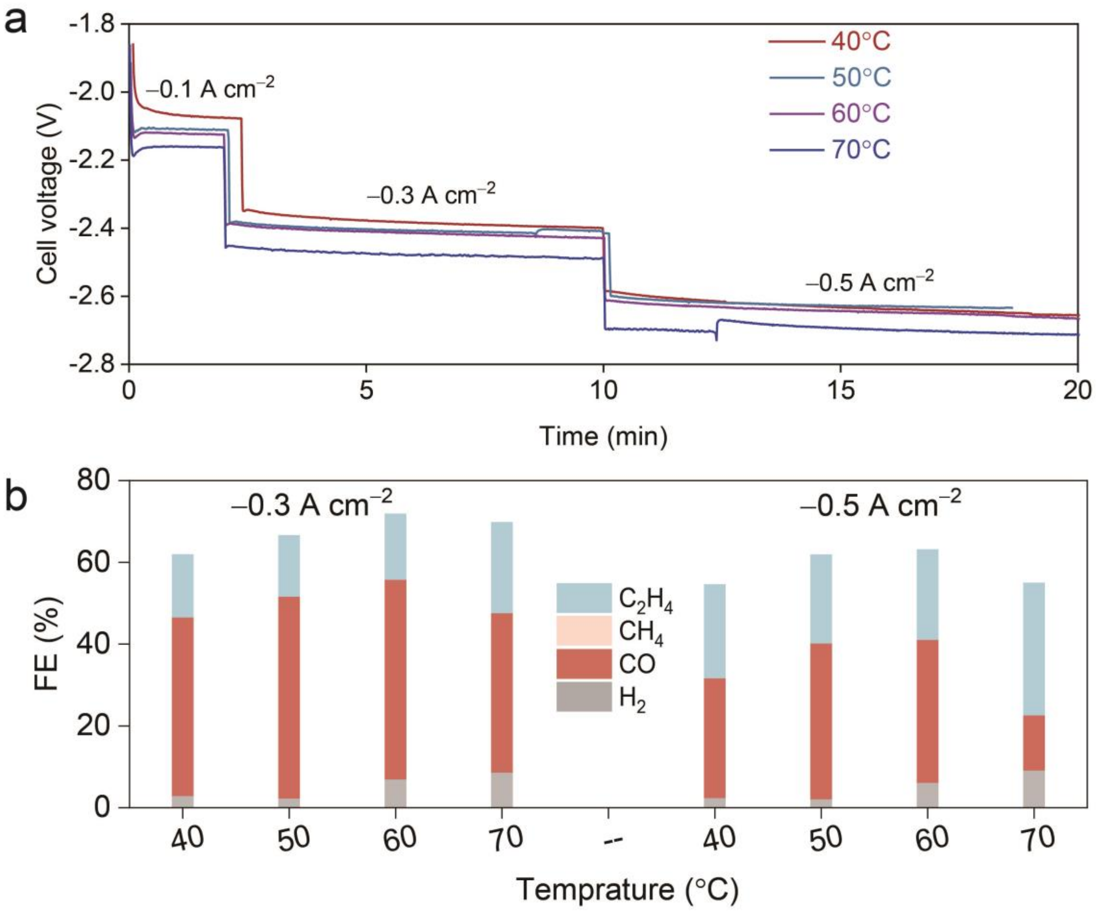

Supplementary Fig. 38. Influence of inlet CO_2 humidification on CO_2R performance with a MEA. **a**, Full cell voltage on the function of the applied currents with different water vapor concentration; **b**, CO_2R products distribution with the variation of inlet water vapor concentration. This water vapor concentration was regulated by heating the gas inlet water container. 1 M KOH was used as an analyte in this experiment.

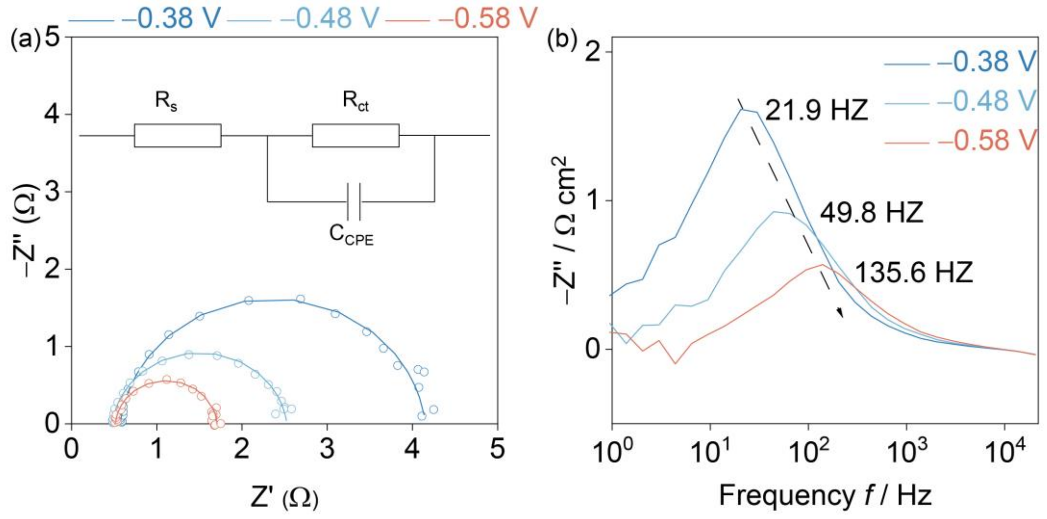

Supplementary Fig. 39. **EIS analysis**. **a**, Nyquist plots of electrochemical impedance spectroscopy for PT/Cu at different applied potentials in 2 M KOH with flow cell, **b**, imaginary part vs. frequency plot with potential as the varying parameter.

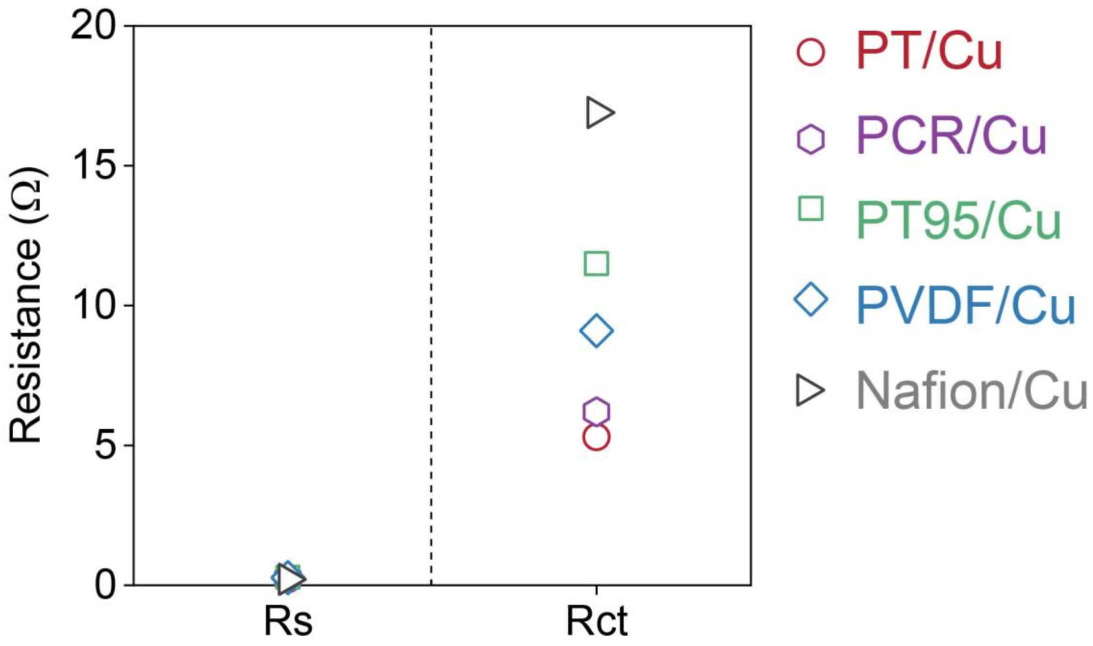

Supplementary Fig. 40. Fitted resistances from Nyquist plots for polymer/Cu GDEs.

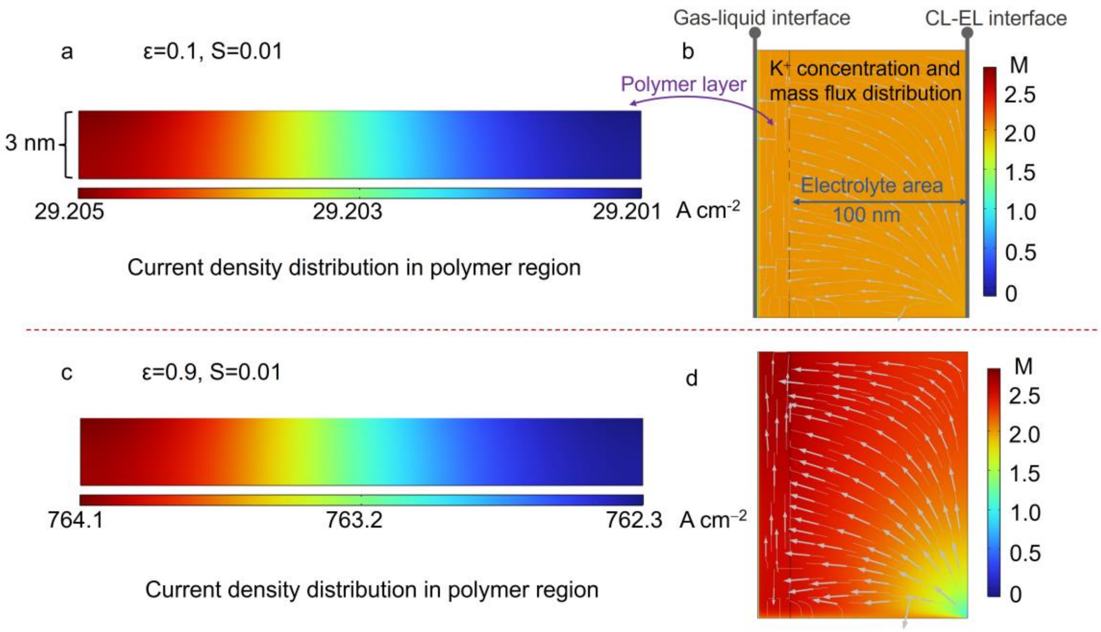

Supplementary Fig. 41. Simulated current density distribution and K^+ diffusion analysis. a, Current density distribution in polymer region and **b**, K^+ concentration and mass flux distribution in the simulated area with polymer porosity as 0.1, polymer layer saturation as 0.01; **c**, Current density distribution in polymer region and **d**, K^+ concentration and mass flux distribution in the simulated area with polymer porosity as 0.9, polymer layer saturation as 0.01. It is observed that the current density distribution within the polymer layer is uniform. Notably, a higher polymer porosity correlates with greater local K^+ availability, leading to increased reaction current density. The direction of arrows in Fig. b and d indicates the diffusion path of K^+.

Note: To ensure that species other than CO_2 in the polymer do not become limiting factors in current transmission, we further calculated the limiting current density under extreme conditions of minimal water content in the polymer. For K^+ is the most abundant cation in the solution and primarily facilitates the conduction of cathodic current, we simplified the conditions for limiting current density to occur when the concentration of K^+ at the electrode surface is zero. The saturation of the polymer region was set to 0.01, with other boundary conditions remaining constant. A steady-state study was performed to simulate this limiting mass transfer problem.

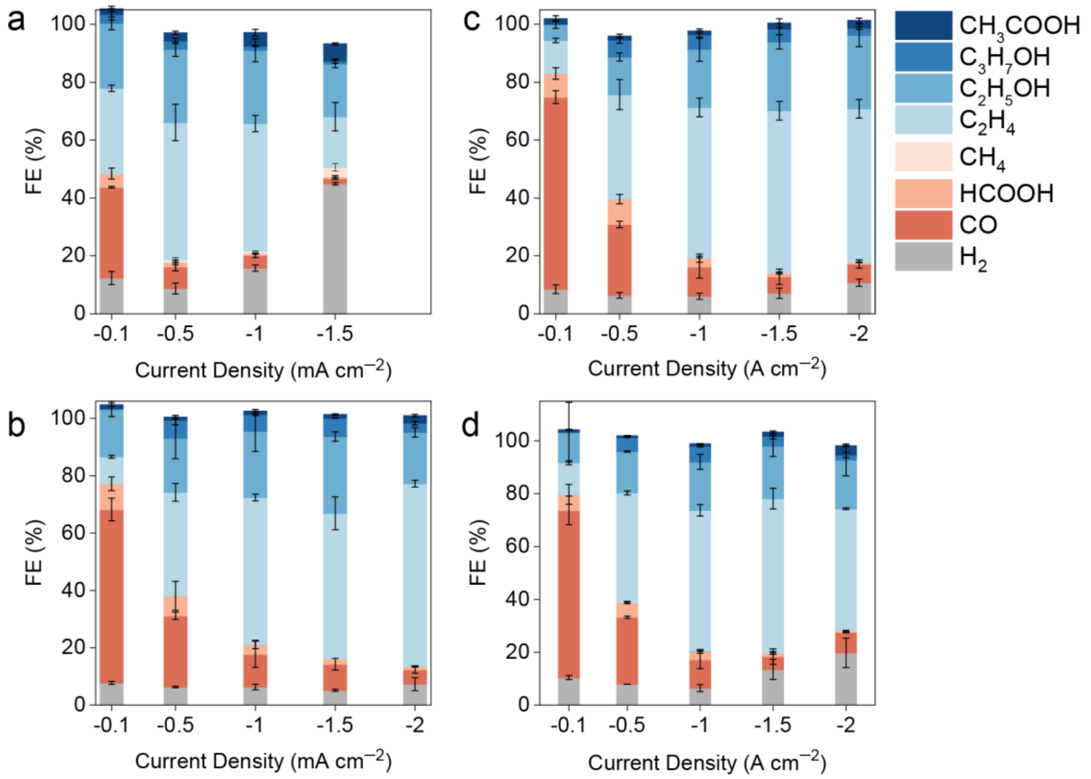

Supplementary Fig. 42. **Products distribution of PT/Cu with different PT loadings**. **a**, 5 µL, **b**, 20 µL, **c**, 40 µL and **d**, 80 µL. The error bars represent standard deviations from at least three independent measurements. Relevant source data are provided in Supplementary Table 17.

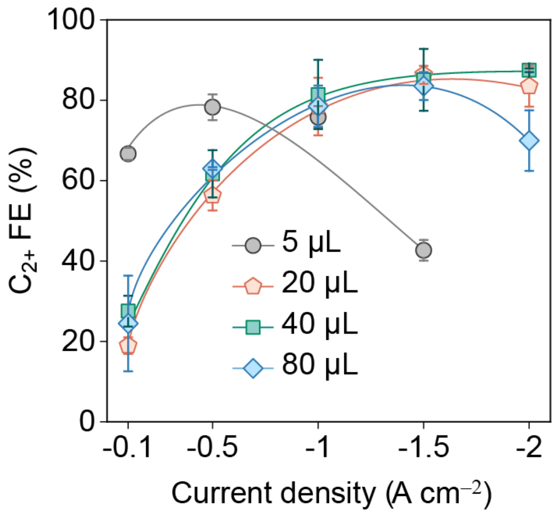

Supplementary Fig. 43. FE_{C2+} on the function of PT loading. The error bars represent standard deviations from at least three independent measurements. Relevant source data are provided in Supplementary Table 17.

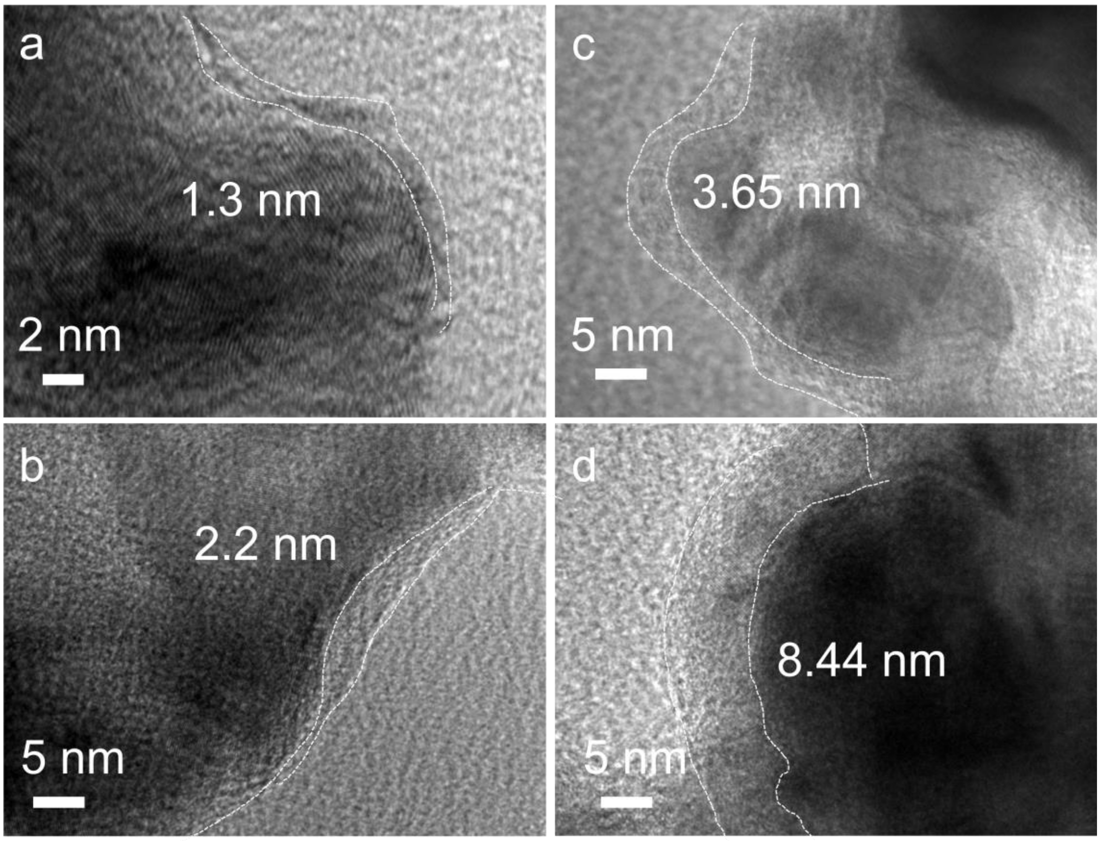

Supplementary Fig. 44. **FETEM images with varied PT polymer loading**. **a**, 5 µL, **b**, 20 µL, **c**, 40 µL and **d**, 80 µL.

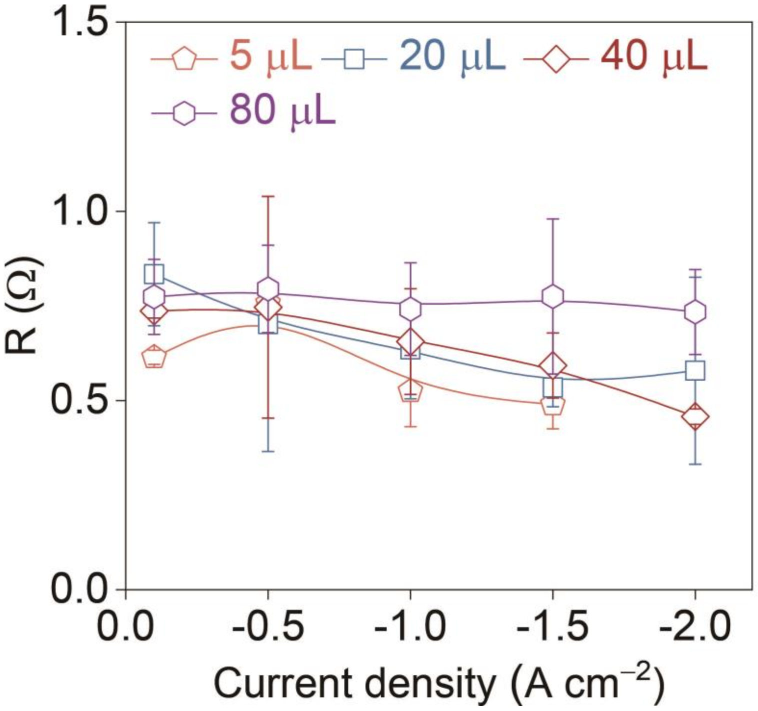

Supplementary Fig. 45. Solution resistance of PT/Cu with varied PT polymer loading. The error bars represent standard deviations from at least three independent measurements.

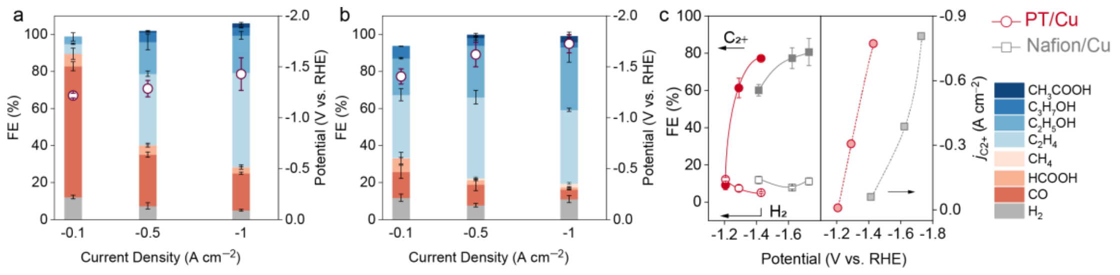

Supplementary Fig. 46. **Products distribution in acid electrolyte**. **a**, PT/Cu and **b**, Nafion/Cu; **c**, comparison of FE_{C2+} and j_{C2+} under identical cathodic potentials for PT/Cu and Nafion/Cu with acid electrolyte. The acid electrolyte was composed of 0.6 M K_2SO_4 and H_2SO_4 (used to adjust the pH to 1.5). The error bars represent standard deviations from at least three independent measurements. Relevant source data are provided in Supplementary Table 18.

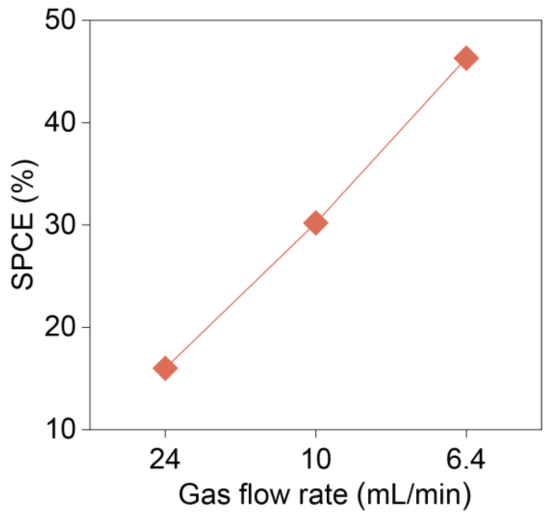

Supplementary Fig. 47. SPCE of CO_2R products on PT/Cu electrode at different CO_2 gas flow rate at a current density of –1 A cm^{-2} with an acidic electrolyte. This electrolyte consisted of 0.6 M K_2SO_4, with H_2SO_4 added to adjust the pH to 1.5.

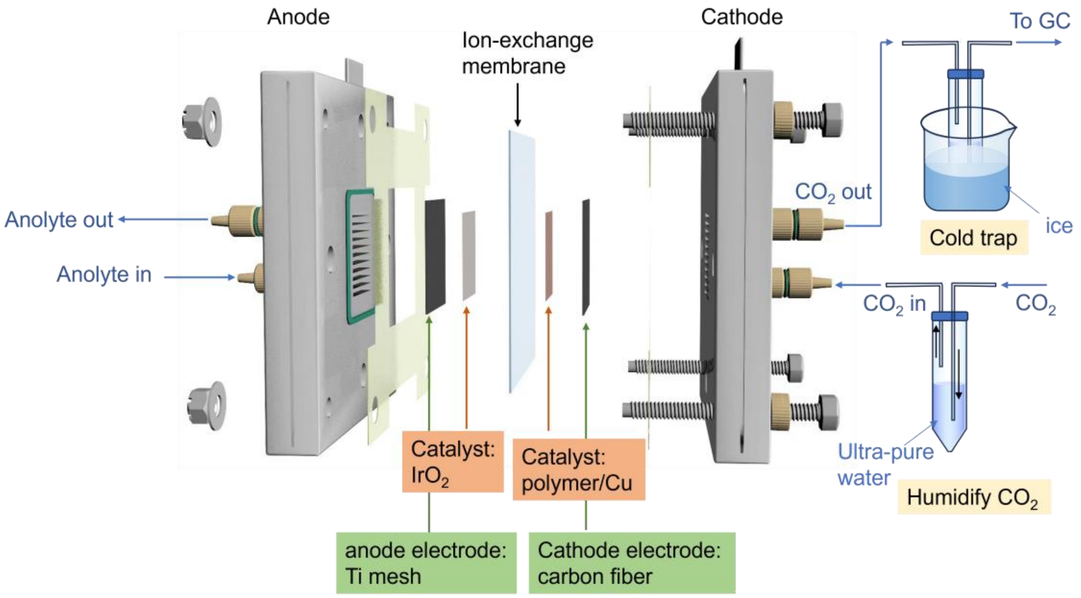

Supplementary Fig. 48. Scheme of membrane electrode assembly.

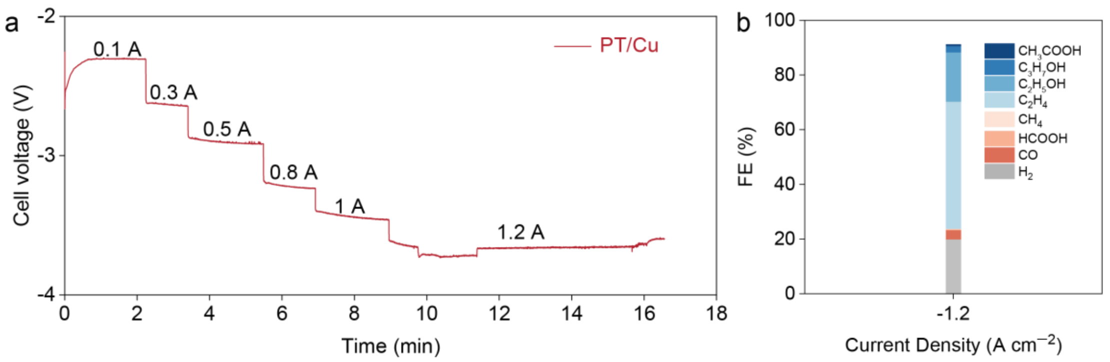

Supplementary Fig. 49. **MEA performances for PT/Cu**. **a**, full cell voltage on the function of the applied current density, **b**, CO_2 products distribution at the current density of –1.2 A cm^{-2}. Test conditions: 1 M KOH as anolyte, the CO_2 flowrate in the cathode is 24 sccm. Before flowing into the MEA cell, the CO_2 is passed through a sealed water container to humidify it.

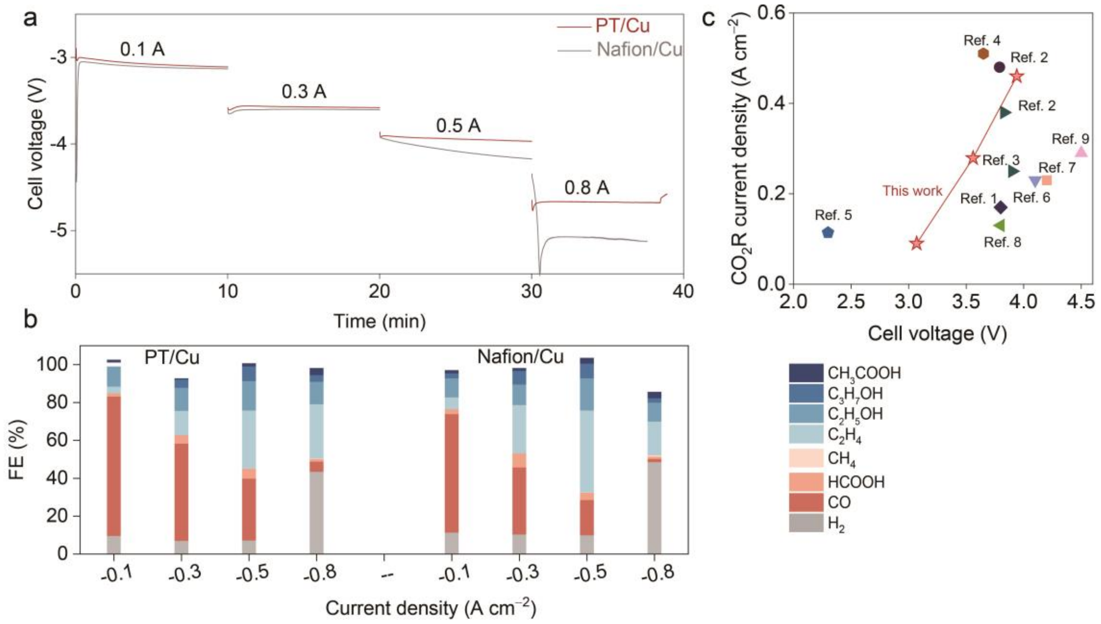

Supplementary Fig. 50. **PT coating for MEA reactor and comparison with other works**. **a**, Full cell voltage on the function of the applied currents. **b**, CO_2R products distribution of PT/Cu and Nafion/Cu. **c**, Comparison of Cell voltage/CO_2R current density between PT/Cu and state-of-the-art Cu-based MEA systems. In this MEA test, 0.1 M $KHCO_3$ was used as the anolyte with a flowrate of 3 sccm. The CO_2 flowrate in the cathode side is 24 sccm. Before introducing CO_2 into the MEA cell, it is passed through a sealed water container for preliminary humidification. The citations in panel c are detailed in Supplementary Table 20.

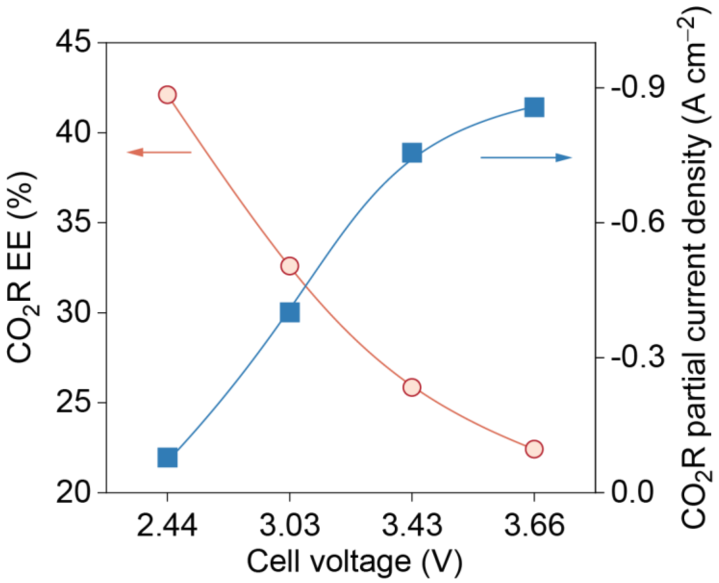

Supplementary Fig. 51. CO_2R EE versus full cell voltage in PT/Cu based MEA. Electrolyte: 1 M KOH.

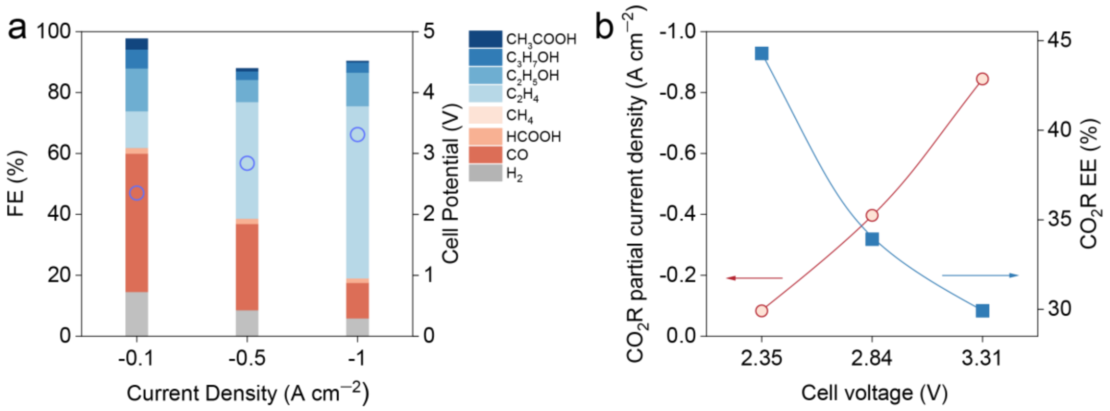

Supplementary Fig. 52. **MEA performance for PT/Cu with 5 M KOH as the electrolyte**. **a**, CO_2R products distribution, **b**, CO_2R partial current density and EE versus cell voltage.

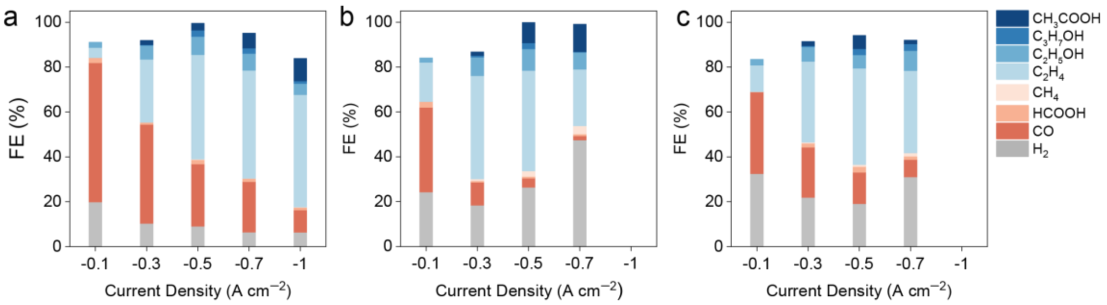

Supplementary Fig. 53. **CO_2R performance in acid electrolyte with a MEA electrolyser**. CO_2R products distribution of **a**, PT/Cu, **b**, Sustanion/Cu, and **c**, Nafion/Cu. Test conditions: 0.6 M K_2SO_4+ H_2SO_4 (regulate pH to 1.5) as the analyte and the flowrate of this electrolyte was set as 3 sccm. CO_2 flowrate in the cathode is 24 sccm.

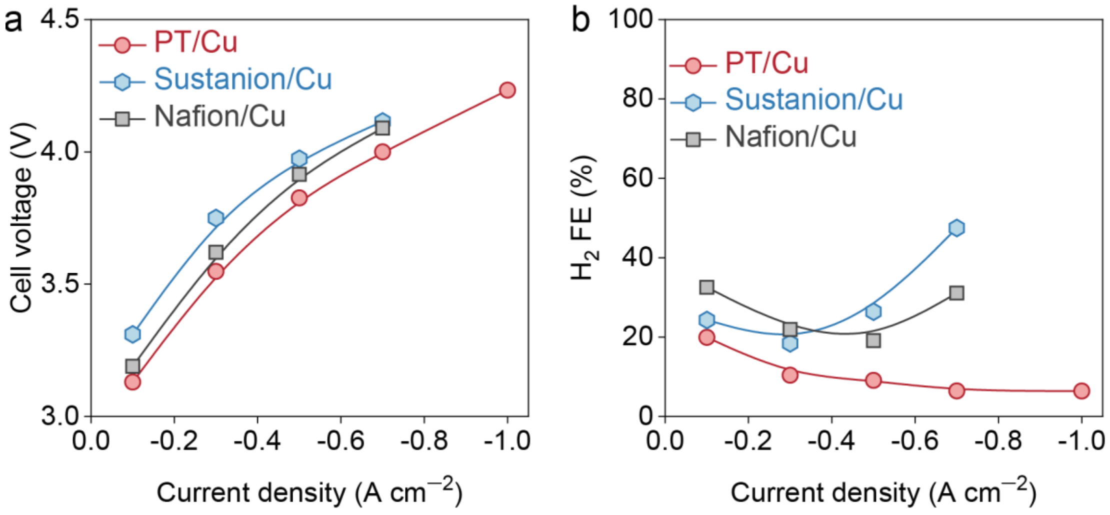

Supplementary Fig. 54. **CO_2R performance comparison in acid electrolyte with a MEA**. **a**, Cell voltage and **b**, H_2 selectivity against current density in acid electrolyte with a MEA electrolyser.

Supplementary Tables

Supplementary Table 1. The modeling parameters with their associated values.

Parameter	Value	Ref.
S_1	-96.31 J mol^{-1} K^{-1}	16
S_2	-148.1 J mol^{-1} K^{-1}	16
S_w	-80.66 J mol^{-1} K^{-1}	16
H_1	7.64 KJ mol^{-1}	16
H_2	14.85 KJ mol^{-1}	16
H_w	55.84 KJ mol^{-1}	16
k_1	10^{-2} s^{-1}	17
k_2	10^{2} s^{-1}	17
k_3	10^{3} L mol^{-1} s^{-1}	17
k_4	10^{9} L mol^{-1} s^{-1}	17
k_w	10^{-3} L mol^{-1} s^{-1}	17
k	$2*10^{-4}$ m s^{-1}	18
M	44 g mol^{-1}	*
D_{OH^-}	$5.3*10^{-9}$ m^2 s^{-1}	10
$D_{HCO_3^-}$	$2.2*10^{-9}$ m^2 s^{-1}	10
$D_{CO_3^{2-}}$	$0.9*10^{-9}$ m^2 s^{-1}	10
D_{H^+}	$9.3*10^{-9}$ m^2 s^{-1}	10
D_{CO_2}	$1.9*10^{-9}$ m^2 s^{-1}	10
D_{K^+}	$2.0*10^{-9}$ m^2 s^{-1}	10
$D_{CO_2,g}$	$1.6*10^{-5}$ m^2 s^{-1}	1
h_{K^+}	0.0922	1
$h_{HCO_3^-}$	0.0967	1
$h_{G,0,CO_2}$	-0.0172	1

h_T	–0.000338	1
K_0	0.034 M atm^{-1}	*
$c_{K^+}^{eq}$	2 M	*
$c_{OH^-}^{eq}$	2 M	*

Supplementary Table 2. Rate parameters for charge transfer reactions.

**Product**	U_k^{o} **(V)**	$i_{\mathrm{o},k}$ **(mA cm⁻²)**	$\alpha_{\mathrm{c},k}$	$\left(\frac{c_i}{c_i^{\mathrm{ref}}}\right)^{\gamma_{j,k}}$	**Ref.**
H_2	0	$1.0 \times 10^{-2}\exp\left(-\frac{0.01[\mathrm{eV}]\mathrm{pH}}{k_{\mathrm{B}}T}\right)$	0.28	1	13
C_2H_4	0.07	$1.9*10^{-6}$	0.67	$\left(\frac{c_{CO_2}}{1[\mathrm{M}]}\right)^{1.36}$	13
C_2H_5OH	0.08	$1.2*10^{-8}$	0.74	$\left(\frac{c_{CO_2}}{1[\mathrm{M}]}\right)^{0.96}$	13
C_3H_7OH	0.09	$4.9*10^{-9}$	0.75	$\left(\frac{c_{CO_2}}{1[\mathrm{M}]}\right)^{0.96}$	13
$HCOO^-$	−0.02	$2.2*10^{-1}$	0.37	$\left(\frac{c_{CO_2}}{1[\mathrm{M}]}\right)^{2.00}$	13
CO	−0.11	2.6	0.17	$\left(\frac{c_{CO_2}}{1[\mathrm{M}]}\right)^{1.5}$	13

Supplementary Table 3. Structure parameters of the catalyst layer and polymer layers.

Parameter	Value	Ref.
d_{PL}	2.00 nm	*Science* **367**, 661-666 (2020)[1]
ε^{CL}	0.5	*Science* **367**, 661-666 (2020)[1]
ε_{PT610}^{PL}	0.73	CO_2 BET test
ε_{PVDF}^{PL}	0.40	CO_2 BET test
$\varepsilon_{Nafion}^{PL}$	0.30	*J. Electrochem. Soc.* **145**, 2677 (1998)[8]
ε_{PCR}^{PL}	0.137	CO_2 BET test
ε_{PT95}^{PL}	0.066	CO_2 BET test
UPT_{PT}^{PL} *	0.58	Water uptake test
UPT_{PVDF}^{PL}	2.15	Water uptake test
UPT_{Nafion}^{PL}	14.80	Water uptake test
UPT_{PCR}^{PL}	0.4	Water uptake test
UPT_{Pt95}^{PL}	0.26	Water uptake test
ρ_{PT}^{PL}	1.48 g cm^{-3}	measured
ρ_{PVDF}^{PL}	1.63 g cm^{-3}	measured
ρ_{Nafion}^{PL}	1.76 g cm^{-3}	measured
ρ_{PCR}^{PL}	1.88 g cm^{-3}	measured
ρ_{PT95}^{PL}	1.65 g cm^{-3}	measured
H_2O/CO_2 ratio (PT)	0.01	calculated
H_2O/CO_2 ratio (PVDF)	0.10	calculated
H_2O/CO_2 ratio (Nafion)	6.58	calculated
H_2O/CO_2 ratio (PCR)	0.06	calculated
H_2O/CO_2 ratio (PT95)	0.07	calculated

Supplementary Table 4. Water uptake of different polymers immersed in liquid water at 25 °C.

**Polymer**	**Water uptake (H_2O/equivalent) Drying method**										
	Initial (mg)	1[a]	2[b]	3[a]	4[b]	5[a]	6[b]	7[a]	8[b]	9[a]	10[b]
Nafion	178.5	205.1	172.6	204.8	172.2	199.8	172.8	203.1	172.6	203.5	172.8
PVDF	137.4	124.0	95.3	98.1	94.7	96.6	94.6	95.6	94.3	/	/
PT	109.7	109.6	107.8	108.1	107.5	108.0	107.2	108.4	106.6	106.5	106.0
PCR	146.1	/	146.0	146.2	145.4	145.6	145.0	145.6	144.6	144.8	/
PT95	144.4	144.6	144.3	144.6	144.0	144.3	143.9	/	/	/	/

a. Polymer immersed in ultrapure water for 24 h (rehydration).

b. Dried at 80°C for 24 h in an oven.

Supplementary Table 5. Calculated water uptake capacity of different polymer membranes.

Polymer	Polymer water uptake (wt%)										
	1	2	3	4	5	6	7	8	9	10	average
Nafion	12.97	15.85	15.72	15.92	13.8	13.51	14.9	15	15.19	15.12	14.80
PVDF	9.73	23.18	2.88	3.47	1.99	1.09	1.31	/	/	/	2.14
PT	1.68	0.29	0.56	0.4	0.66	1.07	1.68	0.53	/	/	0.58
PT95	0.22	0.24	0.4	0.17	0.26	/	/	/	/	/	0.26
PCR	0.16	0.55	0.12	0.41	0.45	0.7	/	/	/	/	0.40

The shaded data are not included in the average calculation due to its large deviation.

Supplementary Table 6. Polymer loading calculation.

**Sample name**	**Catalyst amount (mg)**	**Polymer added (μL)**	**Polymer weight ratio in solvent (mg/μL)**	**Polymer loading (mg/mg_{Cu})**
Nafion/Cu	15	75	0.04184	0.21
PT/Cu	15	40	0.01595	0.04
PTFE/Cu	15	40	0.01775	0.05
PVDF/Cu	15	60	0.01068	0.04
PCR/Cu	15	40	0.01903	0.05
PT95/Cu	15	40	0.0179	0.05

Supplementary Table 7. CO_2R electrochemical data for PT/Cu with flow cell in 2 M KOH. Adapted from the plot of Supplementary Fig. 22a.

***j* (A cm^{-2})**	−0.1	−0.5	−1	−1.5	−2
H_2	7.7	6.3	6.3	5.1	7.3
Error bar	0.5	0.2	1.0	0.4	2.3
CO	60.5	24.8	11.4	9.1	5
Error bar	4.0	1.3	4.6	2	1.2
CH_4	0	0	0	0	0
Error bar	0	0	0	0	0
C_2H_4	9.4	36.2	51.3	50.7	63.7
Error bar	0.5	3.1	1.2	5.7	1.2
EtOH	12.2	18.9	23.1	26.8	17.9
Error bar	5.1	7.2	7	1.6	1.7
PrOH	12.2	18.9	23.1	26.8	17.9
Error bar	5.1	7.2	7	1.6	1.7
Acetate	1.5	1	1.2	1.2	2.6
Error bar	0.6	0.6	0.6	0.3	0.5
Formic acid	9	2.4	9	2.4	9.0
Error bar	6.9	5.1	6.9	5.1	6.9

Supplementary Table 8. CO_2R electrochemical data for PCR/Cu with flow cell in 2 M KOH. Adapted from the plot of Supplementary Fig. 22b.

j **(A cm^{-2})**	−0.1	−0.5	−1	−1.5
H_2	7.0	4.4	4.6	23.3
Error bar	0.2	0.1	0.5	0.2
CO	47.6	14.6	7.4	4.7
Error bar	1.5	0.2	0.3	0.2
CH_4	0.0	0.1	0.1	1.9
Error bar	0.0	0.0	0.0	0.7
C_2H_4	20.3	41.9	44.9	21.3
Error bar	2.1	0.8	1.3	1.6
EtOH	12.0	21.5	33.2	23.4
Error bar	1.9	1.9	2.1	1.1
PrOH	4.1	5.7	3.1	16.9
Error bar	0.8	1.1	0.6	0.2
Acetate	3.0	2.4	4.8	7.9
Error bar	0.1	0.4	2.2	1.4
Formic acid	7.4	3.1	1.4	2.0
Error bar	0.7	0.2	0.1	0.3

Supplementary Table 9. CO_2R electrochemical data for PT95/Cu with flow cell in 2 M KOH. Adapted from the plot of Supplementary Fig. 22c.

***j* (A cm^{-2})**	−0.1	−0.5	−1
H_2	5.4	3.8	21.7
Error bar	0.5	0.3	4.8
CO	38.5	10.6	5.0
Error bar	3.1	0.5	0.8
CH_4	0.0	0.2	2.8
Error bar	0.0	0.1	4.3
C_2H_4	26.8	45.2	26.7
Error bar	0.9	2.8	8.7
EtOH	15.0	28.9	33.3
Error bar	2.8	3.6	1.6
PrOH	6.6	4.1	1.1
Error bar	1.2	0.7	0.6
Acetate	3.9	3.9	5.7
Error bar	0.8	1.7	1.6
Formic acid	6.6	1.9	0.8
Error bar	1.3	0.3	0.2

Supplementary Table 10. CO_2R electrochemical data for PVDF/Cu with flow cell in 2 M KOH. Adapted from the plot of Supplementary Fig. 22d.

j_{total} **(A cm^{-2})**	−0.1	−0.5	−1	−1.5
H_2	16.2	18.3	40.6	62.4
Error bar	0.9	6.3	9.7	2.5
CO	14.5	4.4	3.1	1.1
Error bar	0.9	1.1	2.3	0.2
CH_4	0.0	0.6	1.3	1.2
Error bar	0.0	0.3	0.3	0
C_2H_4	34.6	40.7	24.1	9.3
Error bar	3.5	4.7	9.9	1.2
EtOH	19.9	27.4	18.6	15.3
Error bar	1.6	2.3	2.6	0.3
PrOH	5.5	2.0	0.7	0.2
Error bar	1.6	0.5	0.2	0.3
Acetate	2.0	3.6	4.1	4.7
Error bar	0.9	0.7	0.6	0.7
Formic acid	5.4	0.8	0.6	0.4
Error bar	1.9	0.5	0.2	0.4

Supplementary Table 11. CO_2R electrochemical data for Nafion/Cu with flow cell in 2 M KOH. Adapted from the plot of Supplementary Fig. 22e.

**j_{total} (A cm^{-2})**	−0.1	−0.5	−1
H_2	19.1	12.4	59.0
Error bar	1.2	0.6	0.1
CO	18.4	11.8	1.8
Error bar	3.4	3.1	0.2
CH_4	0.6	0.5	2.4
Error bar	0.2	0.2	0.4
C_2H_4	27.6	42.3	13.8
Error bar	2.0	2.5	1.5
EtOH	16.8	23.8	15.8
Error bar	2.1	1.5	0.8
PrOH	2.4	3.8	0.3
Error bar	2.1	1.7	0.4
Acetate	2.1	1.6	2.9
Error bar	0.4	0.4	0.6
Formic acid	4.7	3.1	0.6
Error bar	3.2	2.0	0.3

Supplementary Table 12. CO_2R electrochemical data for the plots in Fig. 5b-f.

	j_{total}	FE_{C2+} (%)	j_{C2+} (A cm^{-2})	Cathodic potential (V vs. RHE)	C_{2+} cathodic EE (%)
PT/Cu	–0.1	23.0	–0.02	–0.49	15.3
	–0.5	62.4	–0.31	–0.59	39.1
	–1	81.4	–0.81	–0.62	50.5
	–1.5	85.1	–1.28	–0.65	51.8
	–2	87.4	–1.75	–0.73	51.1
PCR/Cu	–0.1	39.4	–0.04	–0.52	25.7
	–0.5	71.5	–0.36	–0.62	44.2
	–1	86.0	–0.86	–0.65	52.4
	–1.5	70.6	–1.06	–0.74	40.8
PT95/Cu	–0.1	52.2	–0.05	–0.55	33.5
	–0.5	82.1	–0.41	–0.65	50.0
	–1	66.8	–0.67	–0.69	39.7
PVDF/Cu	–0.1	66.2	–0.07	–0.58	39.2
	–0.5	74.9	–0.37	–0.68	44.1
	–1	47.7	–0.48	–0.83	27.2
	–1.5	28.9	–0.40	–1.22	13.7
Nafion/Cu	–0.1	48.9	–0.05	–0.57	31.1
	–0.5	71.4	–0.36	–0.68	42.8
	–1	32.8	–0.33	–0.95	17.2

Supplementary Table 13. CO_2 partial pressure experiment for PT/Cu in Figure 6c and Supplementary Fig. 37.

**PT/Cu**	**CO_2 partial pressure**			
**FE**	**100**	**75**	**50**	**25**
H_2	6.3	7.2	7.7	50.7
Error bar	0.2	0.6	1.2	5.1
CO	24.8	21.6	12.1	2
Error bar	1.3	0.5	0.1	0.3
CH_4	0	0	0.1	0.3
Error bar	0	0	0.1	0.2
C_2H_4	36.2	43.5	53.8	25.9
Error bar	3.1	3.2	2.2	2.8
EtOH	18.9	14.9	17.8	13.5
Error bar	7.2	2.2	4.4	2.1
PrOH	6.3	5.3	2.7	0.3
Error bar	1.6	0.7	1.2	0.6
Acetate	1	0.6	0.5	1.9
Error bar	0.6	0.1	0.7	0.5
Formic acid	6.9	4.1	4.5	0.6
Error bar	5.1	0.8	4.5	1.1
FE C_{2+}	61.7	64.2	74.8	41.9
Error bar	5.9	3.8	4.1	3.6
Potential (V vs. RHE)	–0.593	–0.594	–0.626	–0.720
Error bar	0.05	0.03	0.07	0.05

Supplementary Table 14. CO_2 partial pressure experiment for Nafion/Cu in Figure 6c and Supplementary Fig. 37.

**Nafion/Cu**	**CO_2 partial pressure**			
**FE**	**100**	**75**	**50**	**25**
H_2	12.4	41.1	64.6	/
Error bar	0.6	1.4	5.8	/
CO	11.8	6.7	2.3	/
Error bar	3.1	0.7	1.2	/
CH_4	0.5	2.9	2.0	/
Error bar	0.2	0.5	0.1	/
C_2H_4	42.3	23.3	13.5	/
Error bar	2.5	1.2	1.7	/
EtOH	23.8	15.3	8.6	/
Error bar	1.5	1.4	0.6	/
PrOH	3.8	3.9	0.8	/
Error bar	1.7	0.8	0.2	/
Acetate	1.6	1.4	0.4	/
Error bar	0.4	0.1	0.2	/
Formic acid	3.1	1.4	0.9	/
Error bar	2	0.3	0.6	/
FE C_{2+}	71.4	43.9	27.7	/
Error bar	0.4	3.3	1.9	/
Potential (V vs. RHE)	-0.68	-0.73	-0.87	/
Error bar	0.05	0.07	0.02	/

Supplementary Table 15. EIS fitting resistances for polymer/Cu at varied potentials for the plots of supplementary Fig. 39a.

Cathodic potential (V vs. RHE)	**R_s (Ω)**	**R_{ct} (Ω)**
–0.38	0.57	3.58
–0.48	0.48	2.05
–0.58	0.52	1.19

Note: The PT/Cu electrode tested here is a fresh sample, different from the one used for comparations with other polymer/Cu GDEs in Fig. 6e. Consequently, the values of R_{ct} are not totally the same. We have tested at least three electrodes of each kind of polymer/Cu GDEs. Although the R_{ct} values vary for each electrode, the relative trends remain consistent with those shown in Fig. 6e.

Supplementary Table 16. Varied polymer loading and its thickness.

Polymer	Cu loading (mg)	Volume added (μL)	Polymer thickness (nm)	Polymer loading (wt%)
Nafion	15	75	4.37	20.9
PVDF	15	60	4.19	4.3
PT95	15	60	4.19	7.2
PCR	15	40	4.75	5.1
PT	15	5	1.30 (most surface is exposed outside)	0.05
PT	15	20	2.20 (some surface is exposed outside)	2.1
PT	15	40	3.65	4.2
PT	15	80	8.44	8.4

Supplementary Table 17. CO_2R electrochemical data for the PT/Cu with varied PT loadings in the plots of Supplementary 42.

		Polymer loading (μL)			
	***j* (A cm^{-2})**	5	20	40	80
FE of C_{2+}	−0.1	66.6	18.9	27.5	24.5
	Error bar	1.5	2.1	3.8	11.9
	−0.5	78.2	56.3	61.7	63.0
	Error bar	3.2	3.7	5.9	0.3
	−1	75.8	78.4	81.4	78.5
	Error bar	2.8	7.2	8.6	5.1
	−1.5	42.7	86.3	85.08	83.5
	Error bar	2.6	2.2	7.7	3.5
	−2	/	83.5	87.4	70.0
	Error bar	/	5.1	0.4	7.5

Supplementary Table 18. CO_2R electrochemical data in acid electrolyte for the plots in Supplementary Fig. 46c.

	**Potential (V vs. RHE)**	**FE_{C2+} (%)**	**Error bar**	**FE_{H2+} (%)**	**Error bar**	**j_{C2+} (A cm^{-2})**
PT/Cu	−1.21	9.1	2.4	12.2	1.0	−0.01
	−1.29	61.3	5.2	7.4	1.7	−0.31
	−1.43	77.2	1.8	5.1	0.4	−0.77
Nafion/Cu	−1.41	60.1	3.1	11.8	1.9	−0.06
	−1.62	77.4	5.6	7.8	0.9	−0.39
	−1.73	80.7	7.3	11.1	1.9	−0.81

Supplementary Table 19. CO_2R electrochemical data in MEA for the plots in Figure 7a-b and Supplementary Fig. 49.

	**j_{total} (A cm^{-2})**	**Potential (V vs. RHE)**	**H_2**	**CH_4**	**CO**	**C_2H_4**	**EtOH**	**PrOH**	**Acetate**	**Formic acid**
PT/Cu	−0.1	−2.4	18.0	0	70.3	5.5	2.0	0.2	0.3	0.1
	−0.3	−2.8	/	/	/	/	/	/	/	/
	−0.5	−3.0	9.0	0	35.7	31.8	8.0	2.2	1.2	1.3
	−0.8	−3.3	/	/	/	/	/	/	/	/
	−1	−3.4	5.6	0	11	47.9	11.2	3.1	2.3	0.1
	−1.2	−3.6	19.9	0.2	3.4	46.5	18.0	2.3	0.2	0.5
	−0.5	−3.1	9.7	/	43.1	24.0	7.9	3.5	1.8	0.5
	−0.1	−2.5	/	/	/	/	/	/	/	/
Nafion/Cu	−0.1	−2.6	24.5	0	31.7	13.5	8.6	2.5	23.	0.6
	−0.3	−3.1	/	/	/	/	/	/	/	/
	−0.5	−3.4	26.0	4.2	11.1	36.1	9.1	3.7	3.2	2.3
	−0.8	−3.7	/	/	/	/	/	/	/	/
	−1	−3.8	54.5	4.6	2.5	16.9	6.4	2.4	2.1	1.1
	−0.5	−3.3	53.4	0.8	7.2	2.2	7.8	3.1	1.2	2.7
	−0.1	−2.6	/	/	/	/	/	/	/	/

Supplementary Table 20. The summary of detailed CO_2R results with MEA in Supplementary Fig. 50c.

**Catalyst**	**Electrolyte**	**CO_2R current density (A cm^{-2})**	**Cell voltage (V)**	**Ref.**
PT/Cu	0.1 M $KHCO_3$	0.09	3.07	This work
PT/Cu	0.1 M $KHCO_3$	0.28	3.56	This work
PT/Cu	0.1 M $KHCO_3$	0.46	3.94	This work
Cu on PTFE	0.1 M $KHCO_3$	0.17	3.80	1. *ACS Energy Lett.* **6**, 809-815 (2021)
COF-Cu NP (CCBH)	0.1 M $KHCO_3$	0.48	3.79	2. *Nat. Energy*, 1-12 (2023)
Cu (50 nm)	0.1 M $KHCO_3$	0.38	3.83	2. *Nat. Energy*, 1-12 (2023)
Cu (250 nm)	0.1 M $KHCO_3$	0.25	3.90	3. *Joule* **3**, 2777-2791 (2019)
catalyst/tetrahydro-phenanthrolinium/ionomer (CTPI)	0.1 M $KHCO_3$	0.51	3.65	4. *ACS Energy Lett.* **5**, 2811-2818 (2020).
Cu (100)-rich film	0.1 M $KHCO_3$	0.11	2.3	5. *Nat. Catal.* **12**, 5745 (2020)
Cu (100)	0.15 M $KHCO_3$	0.23	4.1	6. *Nat. Catal.* **3**, 98-106 (2020)
Si-Cu	0.1 M $KHCO_3$	0.23	4.2	7. *Nat. Commun.* **12**, 2808 (2021)
Carbon shell-coated Cu	0.1 M $KHCO_3$	0.13	3.8	8. *Nat. Commun.* **12**, 3765 (2021)
Thiol/Ag-Cu	0.1 M $KHCO_3$	0.29	4.5	9. *Nat. Commun.* **12**, 7210 (2021)

Supplementary Table 21. Adapted CO_2R electrochemical data for the plots in Figure 7c.

**Samples**	j_{total} **(A cm^{-2})**	**Potential (V vs. RHE)**	**FE$_{HCOOH}$ (%)**	**FE$_{CO}$ (%)**	**FE$_{H2}$ (%)**
PT/Sn NP	−0.1	−0.49	81.4	7.05	2.47
	−0.5	−0.55	96.2	6.62	2.64
	−1	−0.70	93.4	4.05	2.61
Nafion/Sn NP	−0.1	−0.59	85.7	9.31	2.43
	−0.5	−0.69	80.8	12.27	4.42
	−1	−0.92	84.3	4.46	16.15

Supplementary Table 22. Adapted CO_2R electrochemical data for the plots in Figure 7d.

**Samples**	**j_{total} (A cm^{-2})**	**Potential (V vs. RHE)**	**FE_{CO} (%)**	**FE_{H2} (%)**
PT/Ag NP	–0.1	–0.57	97.3	1.12
	–0.5	–0.79	94.3	5.68
	–1	–0.86	94.1	7.88
Nafion/Ag NP	–0.1	–0.68	97.1	2.15
	–0.5	–0.90	85.5	10.96
	–1	–2.05	34.7	66.97

References

1 García de Arquer, F. P. *et al.* CO_2 electrolysis to multicarbon products at activities greater than 1 A cm^{-2}. *Science* **367**, 661-666 (2020).

2 Johnson, E. F., Boutin, E., Liu, S. & Haussener, S. Pathways to enhance electrochemical CO_2 reduction identified through direct pore-level modeling. *EES catalysis* **1**, 704-719 (2023).

3 Weng, L.-C., Bell, A. T. & Weber, A. Z. Modeling gas-diffusion electrodes for CO_2 reduction. *Phys. Chem. Chem. Phys.* **20**, 16973-16984 (2018).

4 Nitta, I., Hottinen, T., Himanen, O. & Mikkola, M. Inhomogeneous compression of PEMFC gas diffusion layer: Part I. Experimental. *J. Power Sources* **171**, 26-36 (2007).

5 Ismail, M., Ingham, D., Hughes, K., Ma, L. & Pourkashanian, M. Effective diffusivity of polymer electrolyte fuel cell gas diffusion layers: An overview and numerical study. *Int. J. Hydrogen Energy* **40**, 10994-11010 (2015).

6 Kusoglu, A. & Weber, A. Z. New insights into perfluorinated sulfonic-acid ionomers. *Chem. Rev.* **117**, 987-1104 (2017).

7 Kreuer, K.-D. Proton conductivity: materials and applications. *Chem. Mater.* **8**, 610-641 (1996).

8 Divisek, J. *et al.* A study of capillary porous structure and sorption properties of nafion proton-exchange membranes swollen in water. *J. Electrochem. Soc.* **145**, 2677 (1998).

9 Furmaniak, S., Terzyk, A. P., Gauden, P. A., Harris, P. J. & Kowalczyk, P. The influence of carbon surface oxygen groups on Dubinin–Astakhov equation parameters calculated from CO_2 adsorption isotherm. *J. Phys.: Condens. Matter* **22**, 085003 (2010).

10 Newman, J. & Balsara, N. P. *Electrochemical systems*. (John Wiley & Sons, 2021).

11 Wiesenburg, D. A. & Guinasso Jr, N. L. Equilibrium solubilities of methane, carbon monoxide, and hydrogen in water and sea water. *J. Chem. Eng. Data* **24**, 356-360 (1979).

12 Weisenberger, S. & Schumpe, d. A. Estimation of gas solubilities in salt solutions at temperatures from 273 K to 363 K. *AIChE Journal* **42**, 298-300 (1996).

13 Weng, L.-C., Bell, A. T. & Weber, A. Z. A systematic analysis of Cu-based membrane-electrode assemblies for CO_2 reduction through multiphysics simulation. *Energy Environ. Sci.* **13**, 3592-3606 (2020).

14 Tan, Y. C., Lee, K. B., Song, H. & Oh, J. Modulating local CO_2 concentration as a general strategy for enhancing C–C coupling in CO_2 electroreduction. *Joule* **4**, 1104-1120 (2020).

15 Zils, S. *et al.* 3D visualisation of PEMFC electrode structures using FIB nanotomography. *Fuel Cells* **10**, 966-972 (2010).

16 Weaver, J. & Frederikse, H. Crc handbook of chemistry and physics. *CRC Press, Boca Raton* **76**, 12-156 (1977).

17 Schulz, K. G., Riebesell, U., Rost, B., Thoms, S. & Zeebe, R. Determination of the rate constants for the carbon dioxide to bicarbonate inter-conversion in pH-buffered seawater systems. *Mar. Chem.* **100**, 53-65 (2006).

18 Han, J., Eimer, D. A. & Melaaen, M. C. Liquid phase mass transfer coefficient of carbon dioxide absorption by water droplet. *Energy Procedia* **37**, 1728-1735 (2013).
