# Supplementary material for: Selective and stable CO2 electroreduction at high rates via control of local H2O/CO2 ratio
Source: Nat Commun. 2024 Jul 13;15:5893. doi: 10.1038/s41467-024-50269-1 (PMC11246503; doi:10.1038/s41467-024-50269-1)
Supplement: Supplementary file 3 — Description Of Additional Supplementary File [file 41467_2024_50269_MOESM3_ESM.pdf]

**Description of Additional supplementary files**

**Supplementary Movie 1**: Water contact angle test of the PT95/Cu electrode before electrolysis.

**Supplementary Movie 2**: Water contact angle test of the PCR/Cu electrode before electrolysis.

**Supplementary Movie 3**: Water contact angle test of the PT/Cu electrode before electrolysis.

**Supplementary Movie 4**: Water contact angle test of the PVDF/Cu electrode after electrolysis at

–0.5 A cm–2 for 10 min.

**Supplementary Movie 5**: Gas contact angle test of the PT/Cu electrode after electrolysis at –0.5 A cm–2 for 30 min.

**Supplementary Movie 6**: Gas contact angle test of the PT95/Cu electrode after electrolysis at –0.5 A cm–2 for 30 min.
